# Investigating the Complex Arrhythmic Phenotype Caused by the Gain-of-Function Mutation KCNQ1-G229D

**DOI:** 10.3389/fphys.2019.00259

**Published:** 2019-03-18

**Authors:** Xin Zhou, Alfonso Bueno-Orovio, Richard J. Schilling, Claire Kirkby, Chris Denning, Divya Rajamohan, Kevin Burrage, Andrew Tinker, Blanca Rodriguez, Stephen C. Harmer

**Affiliations:** ^1^Department of Computer Science, British Heart Foundation Centre of Research Excellence, University of Oxford, Oxford, United Kingdom; ^2^St Bartholomew’s Hospital, London, United Kingdom; ^3^Department of Stem Cell Biology, Centre for Biomolecular Sciences, University of Nottingham, Nottingham, United Kingdom; ^4^Australian Research Council Centre of Excellence for Mathematical and Statistical Frontiers, Queensland University of Technology, Brisbane, QLD, Australia; ^5^School of Mathematical Sciences, Queensland University of Technology, Brisbane, QLD, Australia; ^6^The William Harvey Research Institute, Barts and The London School of Medicine and Dentistry, Queen Mary University of London, London, United Kingdom

**Keywords:** KCNQ1, long QT syndrome, gain-of-function, arrhythmia, sinus node, computational biology

## Abstract

The congenital long QT syndrome (LQTS) is a cardiac electrophysiological disorder that can cause sudden cardiac death. LQT1 is a subtype of LQTS caused by mutations in KCNQ1, affecting the slow delayed-rectifier potassium current (*I*_Ks_), which is essential for cardiac repolarization. Paradoxically, gain-of-function mutations in KCNQ1 have been reported to cause borderline QT prolongation, atrial fibrillation (AF), sinus bradycardia, and sudden death, however, the mechanisms are not well understood. The goal of the study is to investigate the ionic, cellular and tissue mechanisms underlying the complex phenotype of a gain-of-function mutation in KCNQ1, c.686G > A (p.G229D) using computer modeling and simulations informed by *in vitro* measurements. Previous studies have shown this mutation to cause AF and borderline QT prolongation. We report a clinical description of a family that carry this mutation and that a member of the family died suddenly during sleep at 21 years old. Using patch-clamp experiments, we confirm that KCNQ1-G229D causes a significant gain in channel function. We introduce the effect of the mutation in populations of atrial, ventricular and sinus node (SN) cell models to investigate mechanisms underlying phenotypic variability. In a population of human atrial and ventricular cell models and tissue, the presence of KCNQ1-G229D predominantly shortens atrial action potential duration (APD). However, in a subset of models, KCNQ1-G229D can act to prolong ventricular APD by up to 7% (19 ms) and underlie depolarization abnormalities, which could promote QT prolongation and conduction delays. Interestingly, APD prolongations were predominantly seen at slow pacing cycle lengths (CL > 1,000 ms), which suggests a greater arrhythmic risk during bradycardia, and is consistent with the observed sudden death during sleep. In a population of human SN cell models, the KCNQ1-G229D mutation results in slow/abnormal sinus rhythm, and we identify that a stronger L-type calcium current enables the SN to be more robust to the mutation. In conclusion, our computational modeling experiments provide novel mechanistic explanations for the observed borderline QT prolongation, and predict that KCNQ1-G229D could underlie SN dysfunction and conduction delays. The mechanisms revealed in the study can potentially inform management and treatment of KCNQ1 gain-of-function mutation carriers.

## Introduction

Long QT Syndrome (LQTS) is a type of cardiac disorder that is often related to syncope and sudden cardiac death. LQT1, which is the most common form of LQTS, is caused by mutations in the KCNQ1 gene, affecting the slow delayed-rectifier repolarizing current (*I*_Ks_) (Barhanin et al., 1996; Sanguinetti et al., 1996). Loss-of-function mutations in KCNQ1 can reduce *I*_Ks_ and underlie the inherited form of long QT syndrome (LQT1) (Wang et al., 1996), while gain-of-function mutations in KCNQ1 can act to increase channel opening, resulting in enhanced *I*_Ks_ (Chen et al., 2003; Hong et al., 2005; Lundby et al., 2007; Das et al., 2009; Bartos et al., 2011, 2013; Ki et al., 2014; Moreno et al., 2015).

Gain-of-function mutations in KCNQ1 associate with complex phenotypes. To date, eight gain-of-function mutations in KCNQ1 have been identified that underlie persistent familial atrial fibrillation (AF) (Hancox et al., 2014; Hasegawa et al., 2014), and four have been reported to cause short QT syndrome type 2 (SQT2) (Bellocq et al., 2004; Hong et al., 2005; Moreno et al., 2015; Wu et al., 2015). Some of these gain-of-function mutations are additionally associated with sinus bradycardia [S140G (Chen et al., 2003), V141M (Hong et al., 2005), R231C (Henrion et al., 2012), V241F (Ki et al., 2014), and F279I (Moreno et al., 2015)], and paradoxically, some KCNQ1 gain-of-function mutations have been linked to QT prolongation (borderline LQT) [S140G (Chen et al., 2003), Q147R (Lundby et al., 2007), R231C (Bartos et al., 2011; Henrion et al., 2012) and R231H (Bartos et al., 2013)]. The mechanisms underlying how certain KCNQ1 gain-of-function mutations cause AF and SQT2 have been revealed by *in silico* studies. In general, the gain in *I*_Ks_ function acts to shorten the refractory period and stabilize re-entrant waves, therefore promoting AF and ventricular arrhythmia (Kharche et al., 2012; Zulfa et al., 2016; Adeniran et al., 2017). In addition, the effects of several gain-of-function KCNQ1 mutations (V141M, R231C, and V241F) on sinus bradycardia have recently been explored using human *in silico* models of the sinus node (SN) (Fabbri et al., 2017; Whittaker et al., 2018). However, the mechanisms that underlie why certain KCNQ1 gain-of-function mutations are associated with borderline LQT and the factors that may explain phenotypic variability remain unclear.

The goal of this study is to investigate the ionic, cellular and tissue mechanisms underlying the complex phenotype of a gain-of-function mutation in KCNQ1, p.G229D (c.686G > A), (KCNQ1-G229D) using human atrial, ventricular and SN models informed by *in vitro* patch-clamp measurements. This mutation was first reported in 2014 in a 16-year-old boy with AF (Hasegawa et al., 2014). Interestingly, after radiofrequency catheter ablation therapy sinus rhythm was maintained, but the boy represented with borderline LQT (Hasegawa et al., 2014). Here, we report the clinical features of members of a British family affected by the same mutation. In addition to AF and borderline LQT, sudden death also happened in this family. By using a population of models approach, we investigate how natural variations in ionic current density could underlie variability in the phenotype of mutation carriers. In particular, we focus on the mechanisms that underlie the associated borderline LQT, which has also been reported for other KCNQ1 gain-of-function mutations but has not been explored. In addition, we investigate potential effects on the SN, based on the SN dysfunction caused by other KCNQ1 gain-of-function mutations.

## Materials and Methods

### Clinical Data and QT Interval Duration Assessment

**FIGURE 1:**
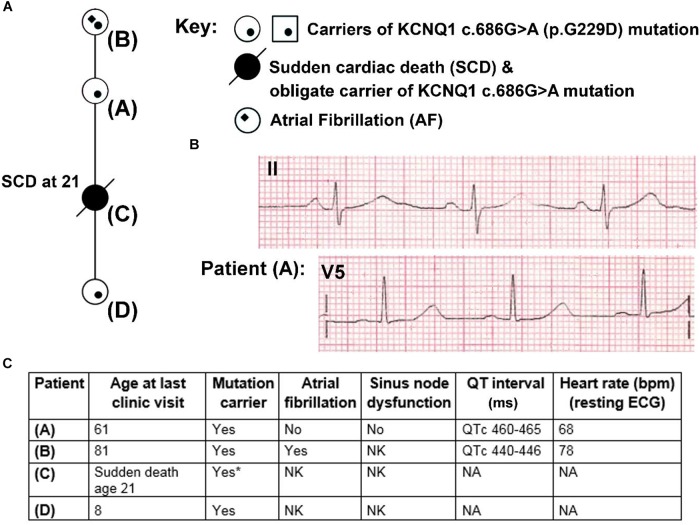
Partial pedigree and clinical information for members of a family carrying the KCNQ1 mutation G229D (c.686G>A). **(A)** Partial mini pedigree of a British family that present with a complex arrhythmic phenotype that includes sudden cardiac death (SCD), atrial fibrillation (AF), and borderline QT prolongation. Circles indicate female family members. **(B)** Lead II and V5 electrocardiograms (ECGs) from Patient (A) with borderline QT prolongation but not AF. **(C)** Clinical characteristics of carriers of the KCNQ1-G229D mutation. ^∗^ = Not genetically tested but obligate carrier of the KCNQ1-G229D mutation (c.686G>A) based on position in the family. Please refer to **(A)** for the location of each patient in the pedigree. NK, not known; NA, not available.

The clinical characterisation of the family carrying the G229D mutation was carried out in accordance with the recommendations of the National Health Service (NHS) Health Research Authority. The protocol was approved by the National Research Ethics Service (NRES Committee) East Midlands - Nottingham 2 [Research Ethics Committee (REC) reference: 09/H0508/74]. All subjects gave written informed consent in accordance with the Declaration of Helsinki. QT interval duration was measured on resting electrocardiograms (ECGs) using lead V5 or II (Figure 1). In patients in sinus rhythm an average of three consecutive beats was calculated. In patients with AF an average of six consecutive beats was calculated. The Bazett formula (Bazett, 1920) was used to correct QT according to heart rate (QTc).

### Molecular Biology and Cell Culture

We characterized the effects of the G229D mutation on KCNQ1/KCNE1 (*I*_Ks_) channel function by whole-cell patch-clamp in a heterologous expression system [Chinese Hamster Ovary-K1 (CHO-K1) cells]. KCNQ1 (GenBank^®^ accession number AF000571) and KCNE1 are as described in Harmer et al. (2014). pEGFP-N1 was from Clontech. The patient-identified G229D mutation (c.686G > A) was introduced into KCNQ1 using site-directed mutagenesis [Quikchange II XL (Agilent Technologies)].

CHO-K1 cells (Sigma-Aldrich, 85051005) were cultured as described in Harmer et al. (2014). To analyze the effects of G229D, cells were transfected with 250 ng of wild-type (WT) KCNQ1 or LQT1 mutant cDNA and 500 ng of KCNE1 (+50 ng pEGFP-N1) (*I*_Ks_-WT or *I*_Ks_-G229D, respectively). To mimic the heterozygous patient phenotype (*I*_Ks_-HET), cells were transfected with 125 ng of wild-type channel + 125 ng of mutant channel and 500 ng KCNE1 (+50 ng pEGFP-N1). Transfections were performed as described in Harmer et al. (2014). After transfection, cells were split at low density onto 10 mm glass coverslips and transfected cells (identified by fluorescence) were patched 48 h later.

### Patch-Clamp Electrophysiological Recording and Analysis

Whole-cell currents were recorded using an Axopatch 200B amplifier (Axon Instruments/Molecular Devices). Data acquisition was performed using pCLAMP10 software through a Digidata 1440A (Axon Instruments/Molecular Devices). Data digitization (sampling) rates were 0.5 kHz and recordings were lowpass Bessel filtered at 1 kHz.

#### Whole-Cell Patch-Clamp

**FIGURE 2:**
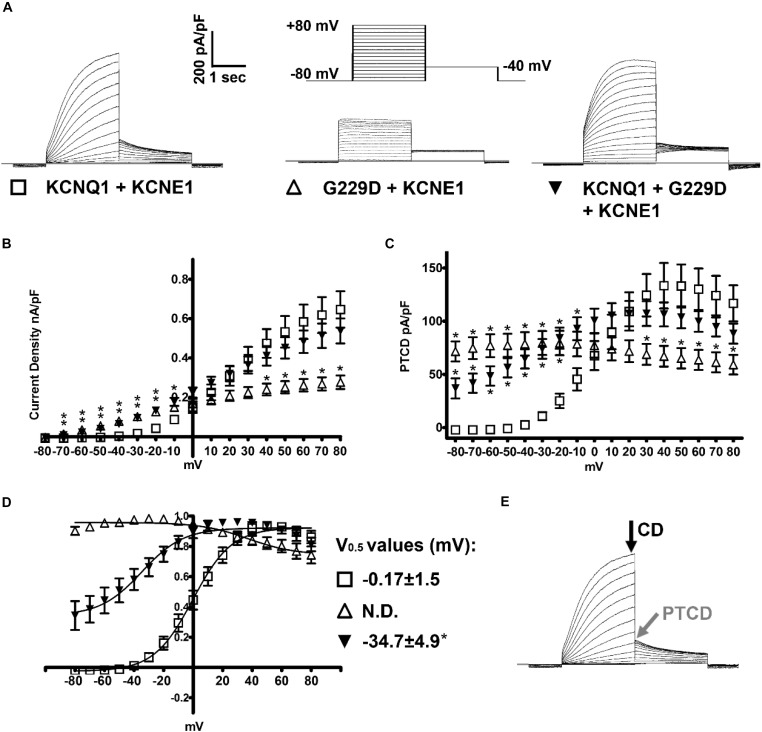
KCNQ1-G229D dramatically alters the biophysical properties of the KCNQ1/KCNE1 (*I*_Ks_) channel. **(A)** Representative traces of the currents produced by wild-type (WT) KCNQ1 (KCNQ1 + KCNE1: *I*_Ks_-WT) or G229D when expressed homozygously (G229D + KCNE1: *I*_Ks_-G229D) or in heterozygous fashion (KCNQ1 + G229D + KCNE1: *I*_Ks_-HET). The effect of the G229D mutation on channel function, in CHO-K1 cells, was analyzed by whole-cell patch-clamp. In all cases, to recapitulate the *I*_Ks_ current, KCNE1 was co-expressed. The zero-current level (0 pA) is indicated by the gray line. The voltage protocol used to elicit these currents is inset in **(A)**. **(B)** Mean current-voltage relationships (current density). **(C)** Peak-tail current density (PTCD). **(D)** Normalized voltage-dependent activation curves (*V*_0.5_) (in mV). The activation curves are fit with Boltzmann functions (solid lines). **(E)** Black and gray arrows indicate the points where the current density signals [current density (CD)] and PTCD were used to calculate the corresponding biomarkers for analysis and fitting. Data are presented as mean ± SEM. (*n* = 8–12). N.D., not determined. ^∗^ indicates significantly different from WT control value (*P* < 0.05) (One-way ANOVA analysis with Bonferroni *post hoc* test).

For the experiments detailed in Figure 2 whole-cell patch-clamp recording was performed at room temperature (22°C) as described in Thomas et al. (2011). The intracellular (pipette) solution contained: (mmol/L) 150 KCl, 10 HEPES, 5 EGTA, 2 MgCl_2_, 1 CaCl_2_ and 5 (Na)_2_ATP (pH 7.2 with KOH). The extracellular (bath) solution contained: (mmol/L) 150 NaCl, 5 KCl, 10 HEPES, 2 MgCl_2_, and 1 CaCl_2_ (pH 7.4 with NaOH). Pipette resistances were, once filled with intracellular solution, between 2 and 3 mega-ohms (MΩ). Pipette capacitance was reduced by coating the pipette tip with SigmaCote (SL2, Sigma). Once the whole-cell configuration had been achieved cells were dialyzed for 2 min before recording. Series resistance (*R*_series_) was compensated by at least 70% using the amplifier circuitry. The liquid junction potential [calculated using the Junction Potential tool in pCLAMP (Axon Instruments/Molecular Devices)] was relatively small (+4.3 mV) and therefore post-recording adjustments of membrane potential were not performed. The voltage protocol used is outlined in Figure 2 and the cycle length for this protocol was 0.1 Hz.

#### Patch-Clamp Recording Analysis

Data were analyzed using Clampfit (Molecular Devices) and GraphPad Prism. As previously described in Thomas et al. (2011) current-voltage relationships were generated by normalizing the maximal current densities at the end of each pulse-potential to cell capacitance. Peak-tail current density (PTCD) was analyzed by normalizing the peak tail currents (in response to the prior test potential) to cell capacitance. The voltage-dependence of channel activation (or steady-state of activation) was determined by fitting the normalized peak tail current amplitudes (*y/y*_max_) versus a test potential (*V*_t_) with a Boltzmann function [*y/y*_max_ = 1/(1 + exp[(*V*_0.5_ -*V*_t_)/*k*])] (*k* indicates the slope factor). The *V*_0.5_ value indicates the potential at which channel activation is half-maximal.

### Computational Modeling of the Effects of the G229D Mutation on KCNQ1/KCNE1 Channel Function

The *I*_Ks_ formulation from the human ventricular O’Hara-Rudy dynamic model (ORd) model (O’Hara et al., 2011) was used to replicate the patch-clamp data (Supplementary Material). Least square curve fitting (lsqcurvefit) was combined with the Multi-Start algorithm in Matlab to find the parameters with optimized fitting results for the mutated *I*_Ks_. Additional fitting details including model formulation are presented in the Supplementary Material. The optimized fitting results for *I*_Ks_-HET (KCNQ1 + G229D + KCNE1) and *I*_Ks_-G229D (G229D + KCNE1) were inserted into the *I*_Ks_ current formulation of the ORd model, the human atrial (Grandi et al., 2011) and (Maleckar et al., 2009) and Fabbri human SN models (Fabbri et al., 2017).

To test whether the effects of our *I*_Ks_-HET formulation on action potential duration (APD) and SN were stable, we also used the *I*_Ks_/*I*_Ks_-HET formulations of Hasegawa et al. (2014) to check the robustness of our results. Action Potential (AP) clamp simulations using three AP traces with different plateau levels were used to examine whether the effect of AP plateau on rapid delayed rectifier potassium current (*I*_Kr_) was model specific by comparing the ORd, Maleckar, and Grandi models.

### *In silico* Populations of Human Ventricular Cell and One-Dimensional (1D) Tissue Fibers Models

A population of 2326 ORd-derived models calibrated with human *in vivo* data was used to account for the effect of human electrophysiological variability as in Zhou et al. (2016). An initial population of 10,000 models was constructed by varying the main ionic conductances by up to ±100% using Latin Hypercube Sampling, including fast sodium current conductance (*G*_Na_), late sodium current conductance (*G*_NaL_), transient outward potassium current conductance (*G*_to_), L-type calcium current conductance (*G*_CaL_), rapid delayed rectifier potassium current conductance (*G*_Kr_), slow delayed rectifier potassium current conductance (*G*_Ks_), inward rectifier potassium current conductance (*G*_K1_), sodium-potassium pump current conductance (*G*_NaK_), sodium-calcium exchange current conductance (*G*_NaCa_), sarcoplasmic reticulum (SR) calcium release permeability (*P*_Jrel_) and SR calcium re-uptake permeability (*P*_Jup_). The initial population of 10,000 models was calibrated using the human *in vivo* measurements described in Zhou et al. (2016). The advantage of using a population of models rather than just a standard baseline model is that it provides scenarios of natural variability (Muszkiewicz et al., 2016), in particular for investigations on multiple disease phenotypes and variable penetrance (Passini et al., 2016).

In the ORd model, the level of *G*_Ks_ is greatest in epicardial cells. Therefore, in order to evaluate the strongest possible effects in ventricles, we simulated the effect of the KCNQ1-G229D mutation in epicardial fibers. A population of monodomain homogeneous epicardial 1D fibers of 2 cm was derived from the ORd single cell population. Pseudo-ECG signals were computed as the integral of spatial gradient of transmembrane potentials from all the points in the fibers (Gima and Rudy, 2002). The tissue simulations and pseudo-ECG calculations were conducted in the open-source software CHASTE (Pitt-Francis et al., 2009) for 50 beats with a conductivity of 3.92 mS/cm to obtain a conduction velocity of 69 cm/s in the baseline ORd epicardial fiber. Transmural fibers consisting of 80% of endocardial cells and 20% of epicardial cells were also simulated for some representative cases with a conductivity of 1.19 mS/cm to obtain a transmural conduction velocity of 40 cm/s in the baseline ORd model.

### Construction and Calibration of Human Atrial Cell Population of Models

Using a similar methodology as in Britton et al. (2013), the nine current conductances of the Grandi atrial cell models (Grandi et al., 2011) were varied by up to ±100% using Latin Hypercube Sampling to generate an initial candidate population of 5,000 models: *G*_Na_, *G*_NaL_, *G*_to_, *G*_CaL_, *G*_Kr_, *G*_Ks_, *G*_K1_, ultrarapid delayed rectifier potassium current conductance (*G*_Kur_), *G*_NaK_, and *G*_NaCa_. These currents were chosen based on their direct contributions to the regulation of APDs, and intracellular calcium fluxes were not varied due to their relatively small effects on APD (Muszkiewicz et al., 2018). After pacing each model under CL = 1,000 ms for 500 beats, the experimental biomarker ranges from human atrial cells were used to select the models in range with the experimental data reported in Sanchez et al. (2014). The models accepted under cycle length (CL) = 1,000 ms were then paced under CL = 2,000 ms and CL = 500 ms. The 917 models that did not display delayed afterdepolarizations, early afterdepolarizations or depolarization failure under all three CLs were accepted for further analysis.

### Construction and Calibration of Human Sinus Node Cell Population of Models

An initial population of 5,000 models was generated from the baseline Fabbri model (Fabbri et al., 2017) by using Latin hypercube sampling to introduce up to ±100% variations to 12 current conductances and ion flux magnitudes: funny current conductance (*G*_f_), *G*_CaL_, T-type calcium current conductance (*G*_CaT_), *G*_Kr_, *G*_Ks_, *G*_to_, *G*_Na_, *G*_NaK_, *G*_NaCa_, *G*_Kur_, *P*_Jrel_, and *P*_Jup_. These currents were chosen because both sarcolemmal currents and calcium handling affect spontaneous depolarization. After simulating each model for 1,000 s, 1046 models with a basic cycle length between 600 and 1,000 ms (heart rate between 60 and 100 bpm) and a positive overshoot membrane potential were accepted for further analysis. The effects of *I*_Ks_-HET in human SN models were classified into three categories: Robust (heart rate between 60 and 100 bpm and a positive overshoot potential), Bradycardia (a positive overshoot potential and heart rate slower than 60 bpm), and Pacemaking failure (a negative maximum potential or a loss of spontaneous activity).

### Statistical Analysis

Patch-clamp experimental data was compared/analyzed using a one-way ANOVA with Bonferroni *post hoc* test for multiple comparisons. Patch-clamp data was considered significantly different if *P* < 0.05. Statistical analysis of *in silico* modeling was conducted with Wilcoxon rank-sum test using Matlab, using a standard *P* < 0.05, and differences in current conductances are reported in the figures and visualized as the differences of the medians of the distributions.

## Results

### Clinical Description of KCNQ1-G229D Mutation Carriers

Patient A was seen after her daughter (Patient C) died unexpectedly whilst sleeping at 21 years of age (Figure 1A). Patient A reported that as a teenager she had occasional periods of fainting but no reported exertional syncope. Her ECG was in sinus rhythm at 68 bpm (Figure 1B,C) and her QTc was 465 ms. It was noted following an ectopic beat that her QTc prolonged to 490 ms.

On the basis of the borderline QT prolongation and the death of her daughter she was genetically tested. Genetic testing found a previously reported pathogenic variant in *KCNQ1* c.686G > A (p.G229D) (Hasegawa et al., 2014). Based on this finding, other members of the family were genetically screened. Screening revealed that her mother (Patient B) and granddaughter (Patient D) are carriers of the *KCNQ1* c.686G > A (p.G229D) mutation. Genetic testing for Patient C was not performed during autopsy but her relationship in the family proves that she was an obligate carrier. Clinical details for Patient D are unavailable. Patient B was first diagnosed with AF at 60 years of age and does not have a history of syncope. Her QTc values, measured in the presence of AF, were 440–446 ms (Figure 1C). Our clinical data, and that reported by (Hasegawa et al., 2014) and (Moreton et al., 2013), indicate that *KCNQ1* c.686G > A (p.G229D) has high penetrance and that it is associated with AF, borderline LQT and sudden cardiac death.

### Effect of the G229D Mutation on *I*_Ks_ Channel Function *in vitro* and *in silico*

Patch clamp measurements show that G229D co-expression with KCNE1 (*I*_Ks_-G229D) produced currents with marked instantaneous activation and tail currents that failed to deactivate (Figure 2A). To mimic the patient phenotype KCNQ1 and G229D were co-expressed (with KCNE1) in heterozygous form (*I*_Ks_-HET). The currents produced by *I*_Ks_-HET possessed both instantaneous and slow activation components reflecting a combined phenotype (Figure 2A) and the presence of G229D acted to shift the voltage-dependence of channel activation (V_0.5_) by approximately -35 mV (Figure 2D).

Overall, our observed effects of G229D on channel function correlate well with the gain-of-function effect first reported by (Hasegawa et al., 2014). Using the electrophysiological data from the patch-clamp studies, we then modeled *in silico* the effects of the G229D mutation on channel function. The fitting details for *I*_Ks_-G229D and *I*_Ks_-HET are shown in Supplementary Figures S1–S3. The resulting *I*_Ks_-G229D and *I*_Ks_-HET models were then incorporated into the populations of human atrial, ventricular and sino-atrial cell models to investigate the complex electrophysiological consequences of the mutation.

### The Predominant Effect of *I*_Ks_ Gain-of-Function G229D Mutation Is APD Shortening in Both the Atria and Ventricle

**FIGURE 3:**
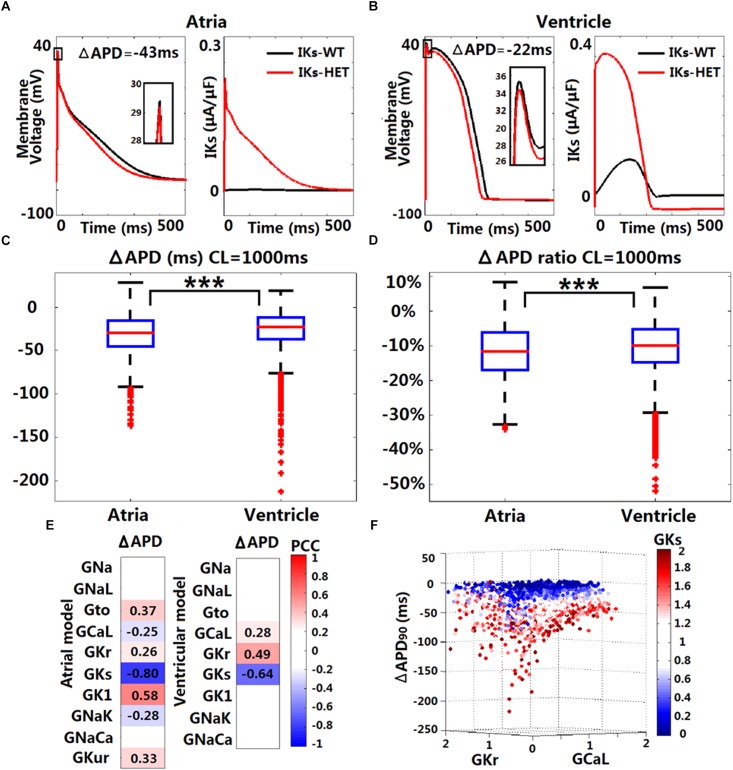
*In silico* simulations of the effects of KCNQ1-G229D on human ventricular and atrial action potentials. The effect of the G229D mutation on membrane voltage (*V*_m_, mV, insets showing peak upstroke) and *I*_Ks_ (μA/μF) in the Grandi human atrial cell model **(A)** and the ORd human ventricular epicardial cell model **(B)**. Comparison of absolute APD change (ΔAPD = APD-*I*_Ks_-HET – APD-*I*_Ks_-WT, **C**) and relative APD change (ΔAPD/APD-*I*_Ks_-WT, **D)** after introducing *I*_Ks_-HET between Grandi atrial population of models and ORd ventricular population of models at CL = 1,000 ms (^∗∗∗^*P* < 0.001) (Wilcoxon rank-sum test). **(E)** Partial correlation analysis between ΔAPD at CL = 1,000 ms and current conductances in the population of atrial and ventricular models. The partial correlation coefficients (PCC) are indicated by the color scale, where red implies a strong positive correlation and blue implies a strong negative correlation. **(F)** Relationship between the conductances of *I*_Kr_, *I*_Ks_, and *I*_CaL_ and the ΔAPD in the ORd population. 0–2 represent the scaling factors for the baseline conductances in the ±100% range.

In the baseline human atrial Grandi model, *I*_Ks_-HET caused significant reductions in APD (14.22%) and weakened the AP upstroke (Figure 3A), in agreement with (Hasegawa et al., 2014). Similarly, in the baseline ventricular ORd model, the presence of *I*_Ks_-HET also weakened the AP upstroke and led to AP shortening by 9.83%. Both APD shortenings occurred because *I*_Ks_-HET produced a much stronger current during the whole AP, and therefore repolarization proceeded more quickly. The degree of shortening in the Grandi atrial model was greater than in the ventricular model (Figure 3A,B), and even greater shortening of APD was seen in the Maleckar human atrial model (33.23% reduction, Supplementary Figure S4). Therefore, the more significant APD shortening observed in human atrial models is not model-dependent.

We investigated potential variability in the effect of *I*_Ks_-HET formulations when inserted in populations of human ventricular and atrial models with variable ionic profiles. As an accumulation of *I*_Ks_ during increases in heart rate may be important for repolarization (Viswanathan et al., 1999), we applied both slow and fast pacing CLs (2,000, 1,000, 500, and 333 ms). For both populations of models, the most common effect of the mutation was APD shortening (Supplementary Figure S5 and Figure 3C,D). Under CL = 1,000 ms, the median APD shortening in the human ventricular cell population was 22 ms, while in the human atrial cell population, the median shortening was 29 ms (Figure 3C,D). Thus, when considering ionic variability in the population, the G229D mutation induced greater APD shortening in human atria than in the ventricular models. Since the baseline Maleckar atrial model already showed an even greater APD shortening than the Grandi atrial model under the mutation, we did not construct a population of Maleckar atrial models to verify this phenomenon. Further analysis showed that the conductances of *I*_Kr_, *I*_Ks_, and *I*_CaL_ were the main determinants for the extent of ventricular APD shortening caused by *I*_Ks_-HET (Figure 3E). Models with weak *G*_CaL_ and *G*_Kr_ and strong *G*_Ks_ tended to present with more significant APD shortening under *I*_Ks_-HET (Figure 3F). In the atrial population of models, a greater number of currents played roles in the regulation of APD shortening, and the most important factor was *G*_Ks_ (Figure 3E). Stronger *G*_Ks_, *G*_NaK_, and *G*_CaL_, and weaker *G*_K1_, *G*_to_, *G*_Kur_, and *G*_Kr_ were associated with more significant APD shortening in the atrial cells (Figure 3E).

### Borderline APD Prolongation May Occur Due to the Interplay Between *I*_Kr_ and *I*_Ks_-HET

**FIGURE 4:**
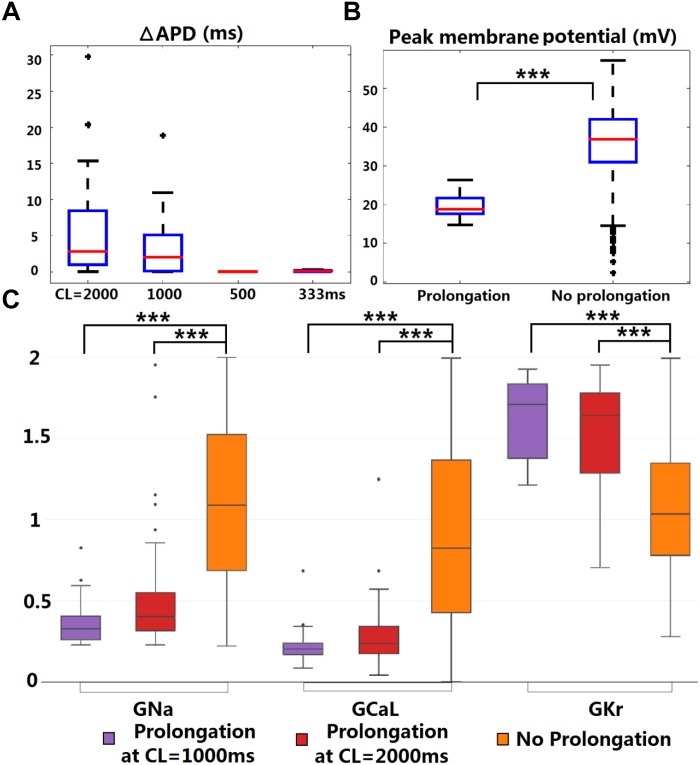
KCNQ1-G229D can lead to ventricular APD prolongation at slow pacing rates. **(A)** APD prolongations under four pacing rates (ΔAPD = APD-*I*_Ks_-HET – APD-*I*_Ks_-WT). **(B)** Comparison of the WT peak membrane voltage between the models that showed or did not show APD prolongation under *I*_Ks_-HET at CL = 1,000 ms. **(C)** Parameter comparison between models that showed APD prolongation at CL = 1,000 or 2,000 ms and those that did not show APD prolongation under *I*_Ks_-HET. The *y*-axis represents the scaling factors in the ±100% range (0–2) to the original baseline ORd model current conductances (^∗∗∗^*P* < 0.001) (Wilcoxon rank-sum test). Black points indicate extreme values that lie more than 1.5 times the interquartile range away from the top (the 75th percentile) or bottom (the 25th percentile) of the box.

Although APD shortening was consistently observed under four pacing CLs (Supplementary Figure S5), some human ventricular models in the population resulted in APD prolongation in the presence of *I*_Ks_-HET, especially at slower pacing rates (Figure 4A). Furthermore, the number of ventricular cell models that showed obvious APD prolongation (>5 ms) was also increased as pacing rates became slower (no models under CL = 500/333 ms, 5 models under CL = 1,000 ms and 25 models under CL = 2,000 ms). Therefore, in the presence of KCNQ1-G229D, APD prolongation occurred more often at slower pacing rates.

There was no significant difference between the WT APDs between the prolongation models and other models in the population. However, the AP peak membrane voltage was significantly reduced (Figure 4B) due to smaller baseline depolarization current conductances (*G*_Na_ and *G*_CaL_) in the models displaying APD prolongation (Figure 4C). In addition, stronger baseline *G*_Kr_ was found in the models displaying APD prolongation at CL = 1,000 or 2,000 ms (Figure 4C). In the subgroup of models producing APD prolongation at CL = 1,000 ms, replacing our *I*_Ks_/*I*_Ks_-HET formulations with the *I*_Ks_/*I*_Ks_-HET formulations of Hasegawa et al. (2014) also generated consistent APD prolongation, supporting the robustness of these phenomena (Supplementary Figure S6).

**FIGURE 5:**
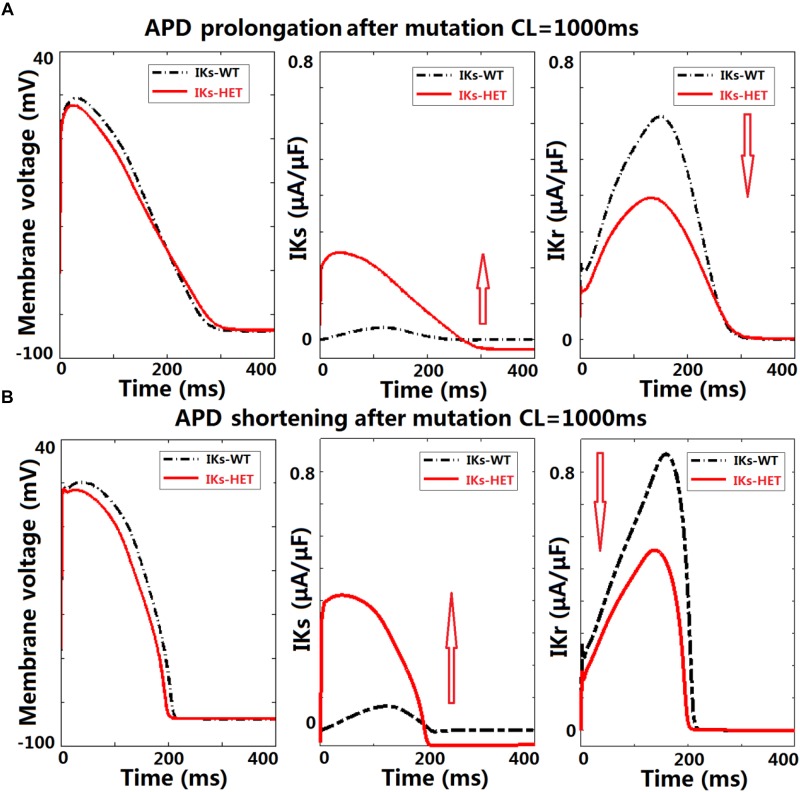
KCNQ1-G229D can lead to ventricular APD prolongation by altering the interplay between *I*_Kr_ and *I*_Ks_. Effects of KCNQ1-G229D mutation (*I*_Ks_-HET) on *I*_Kr_ and *I*_Ks_ in representative models displaying **(A)** APD prolongation and **(B)** APD shortening, at CL = 1,000 ms. The arrows indicate the change of current magnitude after introducing G229D. In **(A)**, the decrease of *I*_Kr_ is more significant than the increase of *I*_Ks_, while in **(B)** the opposite occurs.

To understand the ionic mechanisms underlying APD prolongation/shortening, we analyzed the change of individual currents induced by the presence of the G229D mutation. The biggest differences in ionic currents for both prolongation and shortening were the increase of *I*_Ks_ (Figure 5A,B, middle panels) and the secondary decrease of *I*_Kr_ (Figure 5A,B, right panels). We selected two representative ventricular cell models with similar AP upstroke but one displaying shortening and the other prolongation with *I*_Ks_-HET. The presence of *I*_Ks_-HET affected the AP upstroke and led to a smaller peak membrane voltage and a lower plateau in both models. The reduction in *I*_Kr_ magnitude after G229D introduction was likely due to the reduced phase 2 AP plateau (Figure 5A,B, left panels).

To verify whether this was model-specific, we conducted AP clamp simulations using different human *I*_Kr_ models. The *I*_Kr_ magnitude was consistently weaker under a smaller phase 2 AP plateau in all models tested (Supplementary Figure S7). For the human ventricular model displaying APD prolongation with *I*_Ks_-HET, the decrease of *I*_Kr_ amplitude was slightly bigger than the increase of *I*_Ks_ amplitude under the *I*_Ks_-HET condition (Figure 5A, middle and right panels). In contrast, in the human ventricular model displaying APD shortening, the augmentation of *I*_Ks_ was more significant than the inhibition of *I*_Kr_ (Figure 5B, middle and right panels).

Therefore, our explanation was that if the inhibition of *I*_Kr_ can overcome the augmentation of *I*_Ks_, the presence of the G229D mutation could lead to an overall weaker repolarization, and therefore a prolonged APD. Importantly, the prolongation models tended to have stronger *I*_Kr_ (Figure 4C), which was crucial for *I*_Kr_ reduction to be dominant under *I*_Ks_-HET. We also noticed that under slow pacing, the magnitude of *I*_Ks_ decreased, whereas *I*_Kr_ increased (Supplementary Figure S8), which explained the increased number of models with APD prolongation at slow pacing. Overall, these findings further highlight that in the presence of the G229D mutation, ventricular APD prolongation is more likely to occur during bradycardia, particularly for strong *I*_Kr_ models.

### By Counteracting Action Potential Upstroke Dynamics KCNQ1-G229D Could Promote Tissue Conduction Abnormalities

As illustrated earlier, the G229D mutation can reduce peak AP membrane voltage. We hypothesized that *I*_Ks_-HET by counteracting AP upstroke dynamics (Figure 3A,B, 5A,B) could have important effects on the safety of conduction. In addition, we need to confirm whether the ionic mechanisms underlying APD prolongation in single cells hold true at the tissue level. Therefore, we investigated conduction and repolarization patterns in the presence of *I*_Ks_-HET on the population of human ventricular one-dimensional (1D) fibers.

**FIGURE 6:**
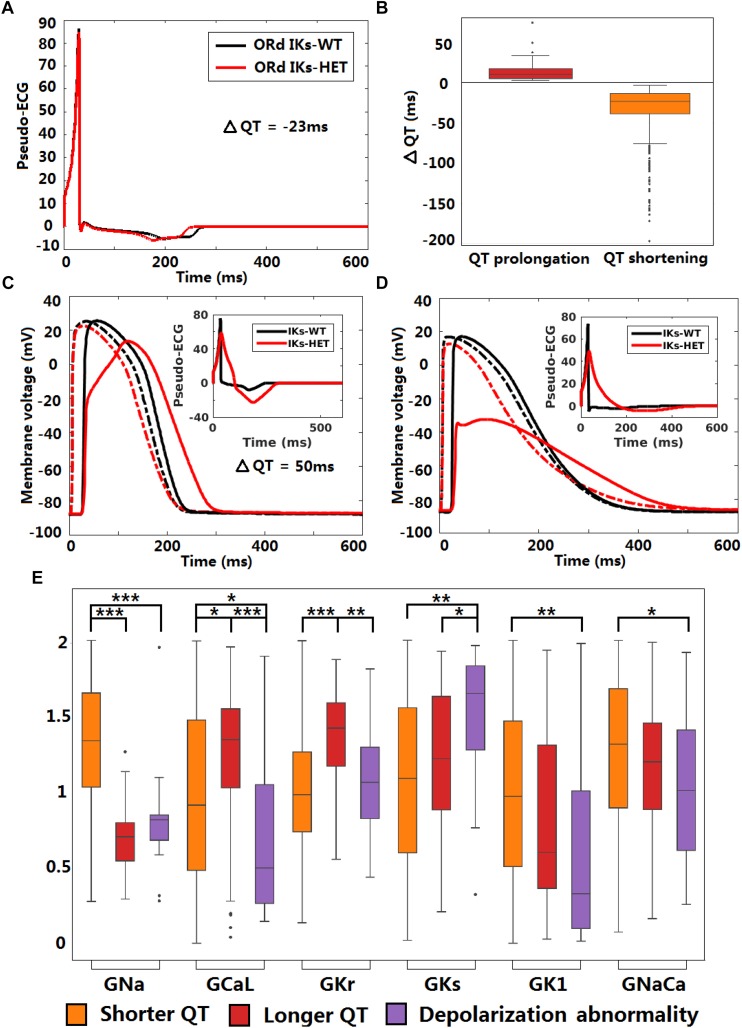
KCNQ1-G229D can impair conduction safety by counteracting action potential upstroke. **(A)** Pseudo-ECG of the original ORd human homogeneous epicardial 1D fiber. **(B)** Longer and shorter QT intervals are possible in the presence of the G229D mutation (*I*_Ks_-HET) in Pseudo-ECGs of the population of human epicardial 1D fiber. **(C)** APs of a fiber that showed slower conduction in the presence of the G229D mutation, with the corresponding pseudo-ECG as an insert. **(D)** APs of a fiber that showed a depolarization abnormality in the presence of the G229D mutation, with the corresponding pseudo-ECG as an insert. **(C,D)** There are 100 nodes in the whole fiber, and Node 20 (dashed lines) and Node 80 (solid lines) are at sites near the beginning and the end of the fiber. **(E)** Comparisons of ionic current conductances between the fibers that showed shorter QT, longer QT and depolarization abnormalities in the presence of G229D (^∗∗∗^*P* < 0.001, ^∗∗^*P* < 0.01, ^∗^*P* < 0.05) (Wilcoxon rank-sum test). Black points indicate extreme values that lie more than 1.5 times the interquartile range away from the top (the 75th percentile) or bottom (the 25th percentile) of the box.

The original ventricular 1D fiber showed a shorter QT interval with the G229D mutation (Figure 6A). In the population of 1D fibers, both significant QT prolongation and QT shortening can be observed (Figure 6B). 36 *I*_Ks_-HET fibers showed QT prolongation compared to the corresponding *I*_Ks_-WT fibers. In the QT prolongation fibers, the AP upstroke was delayed at the end of the *I*_Ks_-HET fiber (Figure 6C). In these cases, the QRS complex was wider, leading to a longer QT interval (Figure 6C, insert). 18 fibers developed depolarization abnormalities under *I*_Ks_-HET, which meant no successful depolarization at the end of the fibers (Figure 6D), and the QT interval was also significantly affected (Figure 6D, insert). Similar results were obtained using transmural fibers (Supplementary Figure S9). By comparing the parameters of the different groups of fibers, we found that the conductances of *I*_Na_, *I*_CaL_, *I*_Kr_, *I*_Ks_, *I*_K1_, *I*_NaCa_ were significantly different. In both QT prolongation and depolarization abnormalities, the baseline *I*_Na_ was weak (Figure 6E). Models exhibiting depolarization abnormalities also tended to have weak baseline *I*_CaL_, *I*_K1_, *I*_NaCa_ and relatively strong *I*_Ks_, which explained the danger of G229D mutation presence in their conduction (Figure 6E). The fibers showing QT prolongation had the strongest baseline *I*_Kr_, which was consistent with the results from the cellular simulations.

### *In silico* Simulations Predict That KCNQ1-G229D Is Capable of Promoting SN Dysfunction by Perturbing Diastolic Depolarization

**FIGURE 7:**
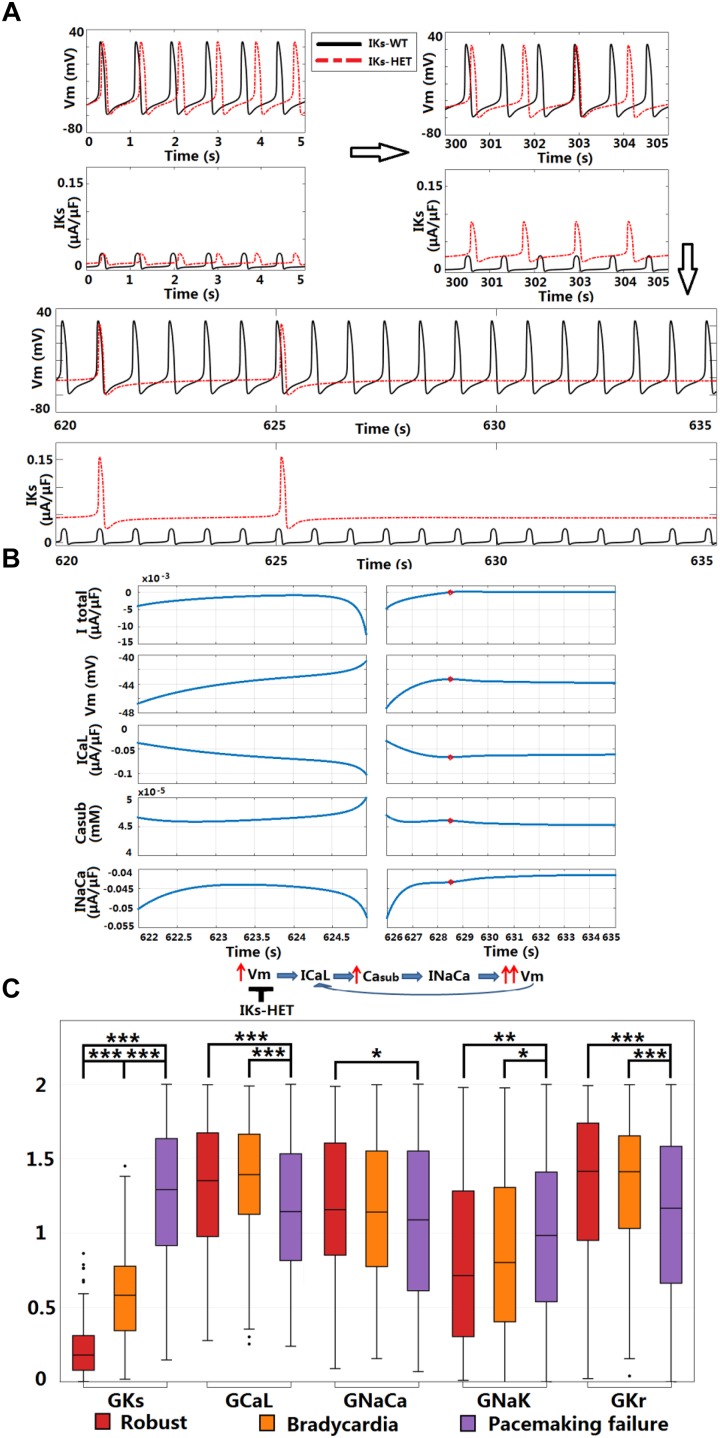
KCNQ1-G229D can cause sinus node dysfunction. **(A)** *I*_Ks_-HET presence results in a loss of sinus rhythm. **(B)** Comparison between the last spontaneous activated beat and the failing process under *I*_Ks_-HET. The red circles in the right columns indicate the time = 628.5 s when diastolic depolarization was interrupted, and membrane potential started to decrease. **(C)** Parameter comparison between Robust, Bradycardia, and Pacemaking failure groups under *I*_Ks_-HET (^∗∗∗^*P* < 0.001, ^∗∗^*P* < 0.01, ^∗^*P* < 0.05) (Wilcoxon rank-sum test). Black points indicate extreme values that lie more than 1.5 times the interquartile range away from the top (the 75th percentile) or bottom (the 25th percentile) of the box.

Sinus node dysfunction and bradycardia has been reported for carriers of different KCNQ1 gain-of-function mutations [S140G (Chen et al., 2003), V141M (Hong et al., 2005), R231C (Henrion et al., 2012), V241F (Ki et al., 2014), and F279I (Moreno et al., 2015)]. Even though SN dysfunction has not been associated with KCNQ1-G229D (Hasegawa et al., 2014) or in the mutation carriers reported here, the effects of V141M on channel gating (Hong et al., 2005) are similar to those induced by the G229D mutation [this study and (Hasegawa et al., 2014)]. Therefore, we investigated whether the G229D mutation can cause SN dysfunction in populations of sino-atrial node cells. In a recently published human SN model (Fabbri et al., 2017), the normal SN model had a stable heart rate (HR) around 73.7 beats per minute (bpm) (Figure 7A). Starting from the same initial condition, when introduced *I*_Ks_-HET produced an increasingly stronger *I*_Ks_ and slower HR, and the sinus rhythm was terminated after 625 s (Figure 7A). Plugging the *I*_Ks_-HET model developed by (Hasegawa et al., 2014) into the simulation was confirmatory, as this model also led to sinus rhythm termination after 135 s (Supplementary Figure S10).

Sinus node activity was related to the interplay between the calcium subsystem and membrane potential in agreement with (Lakatta et al., 2010). For successful spontaneous SN activation, a positive feedback loop between subsarcolemmal calcium (Ca_sub_) and *V*_m_ was needed for the diastolic depolarization. *I*_CaL_ and *I*_NaCa_ provided the biggest depolarization current during the upstroke phase, and the net current excluding *I*_CaL_ and *I*_NaCa_ was always positive (Supplementary Figures S11, S12). The activation of *I*_NaCa_ was regulated by Ca_sub_, and during diastolic depolarization, *I*_CaL_ provided the biggest contribution for the initial accumulation of Ca_sub_ (Supplementary Figure S13). During normal diastolic depolarization, the total net current was inward, leading to very slow/limited activation of *I*_CaL_, accumulation of Ca_sub_ and enhancement of *I*_NaCa_ (Figure 7B, left columns). At the end of diastolic depolarization, the augmentation of *I*_NaCa_ was strong enough to result in a significant increase in *V*_m_ that further activated *I*_CaL,_ promoting faster depolarization in a positive feedback manner to initiate the upstroke phase (Figure 7B, left columns).

In the SN cell model *I*_Ks_-HET produced a much stronger repolarization current to counteract the diastolic depolarization process. At the time of diastolic depolarization interruption (time = 628.5 s), *I*_Ks_ became so strong that the overall total current became outward. The membrane potential then started to decrease, along with the slow decay of *I*_CaL_, Ca_sub_, and *I*_NaCa_ activity (Figure 7B, right columns). Consequently, positive feedback during the depolarization phase was interrupted.

To understand why the carriers of KCNQ1-G229D described in our study and those reported by (Hasegawa et al., 2014) do not present with bradycardia, we used a population of human SN models to explore the effects of heterogeneity in ion channel expression. In the 1,046 human SN cell models, 168 models are robust to *I*_Ks_-HET, 153 models became bradycardic, and the rest (725 models) displayed pacemaking failure. By comparing the parameters, we identified differences in *I*_Ks_, *I*_CaL_, *I*_NaCa_, *I*_NaK_, and *I*_Kr_ conductances between models displaying different phenotypes (Figure 7C). As expected, the Pacemaking failure group had the highest level of *I*_Ks_-HET. A stronger *I*_CaL_ in the Robust and Bradycardia groups can counteract the changes caused by *I*_Ks_-HET and enable safer spontaneous activation. In the Robust group, a stronger inward *I*_NaCa_ and a weaker outward *I*_NaK_ contributed to maintaining negative total current during diastolic depolarization. In addition, a stronger *I*_Kr_ in Robust and Bradycardia groups can counteract the effect of high level *I*_CaL_, preventing excessive APD prolongation (Figure 7C).

## Discussion

In this present study, we investigate the complex phenotypic implications of a gain-of-function mutation in *I*_Ks_ (KCNQ1-G229D) through a combination of computational modeling and simulation and patch clamp experimental characterization, as well as clinical presentation. We describe members of a family that carry KCNQ1-G229D and report that this mutation underlies a complex phenotype characterized by AF, borderline LQT and sudden death. Our clinical findings correlate well with those reported by Moreton et al. (2013) and Hasegawa et al. (2014). We explored the pathogenic role of this mutation using a combination of *in vitro* experiments and *in silico* simulations in human SN, atrial and ventricular models. In addition to providing further evidence supporting the role of G229D in promoting AF as shown in previous studies, we expand our knowledge of G229D and other gain-of-function KCNQ1 mutations in additional ways. Firstly, we present the first mechanistic investigation into why the G229D mutation (and perhaps other KCNQ1 gain-of function mutations) could be associated with a borderline LQT phenotype. Secondly, we demonstrate that the gain-of-function mutation could promote pro-arrhythmic conduction abnormalities by counteracting the AP depolarization phase and reducing conduction safety. This could be a critical mechanism of sudden cardiac death. Thirdly, we utilize populations of human SN models to provide detailed mechanistic predictions which highlight that KCNQ1-G229D could underlie SN dysfunction. Finally, our findings provide plausible reasons for observed phenotypic variability and insights for the clinical management of these patients.

### A Potential Explanation for G229D Associated QT Prolongation

The mechanisms underlying the presence of borderline LQT in G229D carriers [(Moreton et al., 2013; Hasegawa et al., 2014) and this study] and other KCNQ1 gain-of-function mutations (particularly S140G) (Chen et al., 2003; Lundby et al., 2007; Bartos et al., 2011, 2013) are unclear. We used a population of human ventricular cell models to investigate the complex interactions between different currents in the presence of G229D. In addition to APD shortening produced by the standard ventricular model, a subset of the models in the population exhibited APD prolongation. We found that APD prolongation was caused by an interplay between a decrease in *I*_Kr_ activity and increase in *I*_Ks_ activity at slow pacing. Based on our simulations, the instantaneous current component produced by KCNQ1-G229D reduces the magnitude of the AP upstroke which leads to a smaller peak membrane voltage and a lower plateau. Consequently, the presence of a lower plateau acts to decrease the activity of *I*_Kr_ which, in turn, acts to prolong APD. In our fibers showing QT prolongation at the tissue level, *I*_Kr_ tended to be stronger, suggesting that the *I*_Kr_/*I*_Ks_ interplay mechanism, originally identified in single cells, also holds true at the tissue level.

### KCNQ1-G229D May Induce Defects in Conduction

The 1D fiber results also indicate that the presence of KCNQ1-G229D could impair myocardial conduction. Although QRS widening in fibers was not observed clinically in mutation carriers, whole ventricle simulations have shown that QRS width is more sensitive to the activation pattern in the conduction system rather than myocardial propagation (Cardone-Noott et al., 2016). Therefore, local conduction abnormalities in the myocardium may still be present even with normal QRS width. Local or regional conduction abnormalities may also occur due to heterogeneous expression of KCNQ1/G229D throughout the ventricles (Liu and Antzelevitch, 1995; Viswanathan et al., 1999). Although the fiber simulations we used do not account for the full heterogeneity known to span the human ventricles, they do provide a rough approximation of tissue behavior. Despite these potential limitations, our findings emphasize that G229D could enhance regional differences in conduction and this could contribute to the substrate required for the formation of a lethal arrhythmia.

### *In silico* Modeling Using Human Models Provides Explanations for SN and Atrial Dysfunction

Our human SN model simulations predict that the G229D mutation is likely to underlie SN dysfunction and that this could increase the risk of sinus arrest. By examining variations in ionic current density, our population of SN models may also provide plausible explanations as to why a dysfunctional SN phenotype was not seen by Hasegawa et al. (2014) or in the mutation carriers we report. Based on the mechanisms revealed in this study and those of Fabbri et al. (2017) and Whittaker et al. (2018), disturbed SN activity could be a general action of KCNQ1 gain-of-function mutations that alter channel gating in a similar fashion.

Mechanistically, the G229D mutation has been postulated to cause AF by promoting atrial APD shortening (Hasegawa et al., 2014) and two and three-dimensional tissue models have described that this mutation promotes the sustainment of re-entrant waves thereby increasing susceptibility to atrial arrhythmia (Zulfa et al., 2016). In our baseline and population of models, the G229D mutation results in atrial and ventricular AP shortening, but the average degree of shortening is less for ventricular than atrial APs, which agrees with the findings of Hasegawa et al. (2014) and implies a more prominent effect of the mutation on the human atria.

### Clinical Implications for KCNQ1-G229D Carriers

KCNQ1-G229D presents in adults largely as AF, and Class I drugs such as flecainide and quinidine may be prescribed. Based on our simulation results, KCNQ1-G229D could impair conduction by counteracting AP upstroke, and class I sodium channel blockers could exacerbate this. Furthermore, our simulations predict that this mutation could underlie SN dysfunction which has been postulated to act as a substrate for the development of AF (Duhme et al., 2013). Indeed, a trend in disease progression from bradycardia in to persistent AF has been reported for patients that carry the KCNQ1 gain-of-function mutation V241F (Ki et al., 2014). As revealed by our SN simulations, *I*_CaL_ played a crucial role in the maintenance of normal sinus rhythm in the presence of the G229D mutation. Therefore, drugs with class IV calcium channel blocking actions could unravel bradycardia in G229D mutation carriers with normal sinus rhythm.

Our simulations showed that QT prolongation was primarily observed during bradycardia implying that the prevention of bradycardia to maintain sinus rhythm should be considered in the management of mutation carriers. The use of drugs with a negative chronotropic effect, such as beta-blockers, should therefore be reviewed and device implantation considered for KCNQ1 gain-of-function mutation carriers that present with bradycardia.

Another intriguing observation is that some G229D mutation carriers have died suddenly whilst sleeping [reported in this study and (Moreton et al., 2013)]. Sudden cardiac arrest during sleep has also been reported for a carrier of KCNQ1-R231H (Bartos et al., 2013). Unfortunately, we do not have the necessary clinical information to establish the precise mechanisms underlying these deaths. In LQT1, cardiac arrest normally occurs during exercise and sudden cardiac death during sleep is more a feature of LQT3 (Schwartz et al., 2001). Therefore, we can propose two possible mechanisms: sinus arrest without escape rhythms or a lethal arrhythmia caused by severe QT prolongation. It is worth noting that sinus arrest, due to SN dysfunction, is an unusual cause of death and SN disease in the absence of symptoms is not generally considered prognostically important. In view of these considerations we suspect that the most likely mechanism of sudden death in these patients is the promotion of a lethal arrhythmia by QT prolongation and/or conduction block.

### Limitations of the Study

The effects of the G229D mutation on *I*_Ks_ channel function were modeled in a heterologous expression system. Therefore, it is possible that the expression and kinetics of the mutant channel complex could be distinct in cardiomyocytes. We were limited to this model because: (1) it is not possible to use mice or rats as a model as these species do not use *I*_Ks_ for cardiac repolarization in adult life (Nerbonne, 2014). (2) The generation of transgenic rabbit models of KCNQ1 mutations (Brunner et al., 2008), would be prohibitively expensive and the higher heart rate of this species would likely confound modeling the effects of the mutation on the SN. (3) The current utility of human induced pluripotent stem derived cardiomyocytes (hiPSC-CMs) for examining *I*_Ks_ function has been questioned and this may relate to their relative immaturity (Christ et al., 2015). We would also like to highlight that although we propose an explanation for the borderline LQT seen in carriers of the G229D mutation the observed APD prolongations in the population of models subset were relatively mild. This could relate to the potential differences between the function of the mutant channel complex in the heterologous expression system versus in cardiomyocytes or alternatively it could imply that other mechanisms contributing to QT prolongation exist. In the future, the validation of our *in silico* predictions in a physiological system is warranted. hiPSC-CM technology is rapidly advancing, and we hope that in time we will be able to use this model to study the effects of the G229D mutation in human cardiomyocytes that possess adult-like and chamber/region specific electrophysiological properties.

## Conclusion

By using a combined *in vitro* and *in silico* approach we have explored how the KCNQ1 mutation G229D can underlie the reported phenotype of AF and borderline QT prolongation. In addition, our modeling results suggest that the G229D mutation can cause conduction abnormalities, and can underlie SN dysfunction. Importantly, our results suggest that for G229D mutation carriers (and perhaps for other KCNQ1 gain-of-function mutation carriers), the prescription of beta-blockers, class I sodium channel blockers and compounds with class IV calcium channel blocking properties should be used with caution.

## Data Availability

The datasets generated for this study are available on request to the corresponding author.

## Author Contributions

XZ conducted the *in silico* simulations, took part in the design, analysis and interpretation of the modeling results. AB-O contributed to the design, interpretation, and discussion of the *in silico* results. RS, CK, CD, and DR were involved in collation of patient data and clinical interpretation. KB contributed to the design of the model fitting process. BR took part in the design, interpretation, discussion, and provided the funding for the modeling work. AT and SH conducted the *in vitro* experimentation, overviewed the project design, interpretation and discussion, and provided funding for the *in vitro* experiments. All authors contributed to writing the manuscript.

## Conflict of Interest Statement

The authors declare that the research was conducted in the absence of any commercial or financial relationships that could be construed as a potential conflict of interest.
