# Supplementary material for: Investigating the Complex Arrhythmic Phenotype Caused by the Gain-of-Function Mutation KCNQ1-G229D
Source: Front Physiol. 2019 Mar 18;10:259. doi: 10.3389/fphys.2019.00259 (PMC6430739; doi:10.3389/fphys.2019.00259)
Supplement: Supplementary file 1 [file Data_Sheet_1.pdf]

Supplementary Material:

Investigating the complex arrhythmic phenotype caused by the gain-of-function mutation KCNQ1-G229D

Xin Zhou, Alfonso Bueno-Orovio, Richard J. Schilling, Claire Kirkby, Chris Denning, Divya Rajamohan, Kevin Burrage, Andrew Tinker, Blanca Rodriguez and Stephen C. Harmer.

This PDF file includes:
Supplementary text
Figures S1 to S13
References for supplementary material

Supplementary Methods:

Computational modelling of the G229D mutation

The following biomarkers were used in the fitting process (Figure S1): current density (CD), peak-tail current density (PTCD), time of half-activation (Activation Thalf), half activation time after removing the instantaneous activation current (Activation Thalf-Inst), and the time constant of the deactivation trace after fitting the standard exponential equation (Deactivation Tau). In order to capture the effects on channel function of the G229D mutation, another biomarker deactivation steady state (DSS) was extracted from the patch clamp traces as the steady state current density level at the end of the deactivation process.

Since I_{Ks} channels were over-expressed in CHO-K1 cells, current signals from CHO-K1 experiments were much larger than those in human cardiomyocytes and mathematical models. To normalise the large magnitude of I_{Ks} currents in CHO-K1 cells into physiological bounds, we assumed the correlation between CDs of wild-type (WT) I_{Ks} in experiments and those in the ORd model (O'Hara et al. 2011). A good linear correlation was found between the WT CHO-K1 CDs and the WT ORd model CDs. Therefore, CD signals were rescaled before being used in the fitting, according to: ORd model CD= 0.0019*CHO CD+0.0026 (r^2=0.9914). Similarly, peak-tail current density (PTCD) and DSS from CHO-K1 cells were rescaled using the same formula. This inherently takes into account the use of a Q10 factor for the rescaling of current magnitude to physiological temperature.

The ORd I_{Ks} ordinary differential equations (ODEs) were used as the framework in the fitting process to the CHO-K1 patch clamp results. X1 and X2 are the two gates of I_{Ks}, and Xss is their steady state, with Tau1 and Tau2 being the time constants (rate of changes) of the two gates, respectively. During the in silico patch clamp experiment, the same voltage step protocol used experimentally was applied to the I_{Ks} model as the input values of transmembrane potential (V_m), and the ODEs of the two I_{Ks} gates were solved using the MATLAB ODE solver ode15s, to produce the in silico I_{Ks} traces.

For WT, HET and G229D fitting, the structure of I_{Ks} ODEs is the same as the original ORd model:

$$\frac{dX1}{dt} = \frac{Xss - X1}{Tau1}$$

$$\frac{dX2}{dt} = \frac{Xss - X2}{Tau2}$$

$$I_{Ks} = G_{Ks} \cdot \left(1 + \frac{0.6}{1 + \left(\frac{3.8 \cdot 10^{-5}}{Ca^{2+}{}_i}\right)^{1.4}} \right) \cdot X1 \cdot X2 \cdot (V_m - E_{Ks})$$

Equation legend. X1 and X2 are the two gates of I_{Ks}. Xss is the steady state of both gates, and Tau1 and Tau2 are the time constants of these gates, respectively. G_{Ks} is the conductance of I_{Ks}, and $Ca^{2+}{}_i$ is the intracellular calcium concentration, and E_{Ks} is the reversal potential for I_{Ks}.

The same patch clamp biomarkers used in experiments were calculated from the simulated I_{Ks} traces. By varying the parameters in Xss, Tau1 and Tau2, the parameter sets which produced the best fitting with experiments are the following:

For wild type:

$$Xss\_WT = \frac{1}{1 + e^{\frac{-(Vm+30.1874)}{10.5108}}}$$

$$\text{Tau1\_WT} = 749.1159 + \frac{1}{0.000031974 \cdot \exp^{\frac{\text{Vm}+61.1291}{10.8357}} + 0.000067085 \cdot \exp^{\frac{-(\text{Vm}+203.1010)}{232.2352}}}$$

$$\text{Tau2\_WT} = \frac{1}{0.0019 \cdot \exp^{\frac{\text{Vm}-44.7948}{17.7931}} + 0.0011 \cdot \exp^{\frac{-(\text{Vm}+66.0925)}{30.0977}}}$$

For heterozygous mutation:

$$\text{Xss\_HET} = \frac{0.9465}{1 + e^{\frac{-(\text{Vm}+71.1239)}{27.4914}}}$$

$$\text{Tau1\_HET} = 376.3354 + \frac{1}{0.000041329 \cdot \exp^{\frac{\text{Vm}+70.7755}{11.4172}} + 0.000098594 \cdot \exp^{\frac{-(\text{Vm}+202.6051)}{97.8909}}}$$

$$\text{Tau2\_HET} = \frac{1}{0.0019 \cdot \exp^{\frac{\text{Vm}-87.0654}{106.6171}} + 0.000031856 \cdot \exp^{\frac{-(\text{Vm}+355.0597)}{157.0970}}}$$

For homozygous mutation:

$$\text{Xss\_G229D} = \frac{0.7029}{1 + e^{\frac{-(\text{Vm}+226.8211)}{38.7274}}}$$

$$\text{Tau1\_G229D} = 196.0892 + \frac{1}{67.7032 \cdot \exp^{\frac{\text{Vm}+157.7247}{14.8435}} + 94.1943 \cdot \exp^{\frac{-(\text{Vm}+458.5436)}{169.4608}}}$$

$$\text{Tau2\_G229D} = \frac{1}{3.7679\text{e}(-14) \cdot \exp^{\frac{\text{Vm}-201.2472}{126.1263}} + 0.00062011 \cdot \exp^{\frac{-(\text{Vm}+34.5247)}{91.2525}}}$$

The fitting results are shown in Figure S2. The action potentials obtained with the original ORd I_{Ks} formulation and the new fitting with the WT data are nearly identical (Figure S3). Since the ORd formulation was constructed and validated based on recordings in human ventricular cardiomyocytes, we chose to use this formulation for the WT rather than the new formulation fitted to CHO-K1 data.

Supplementary Figures:

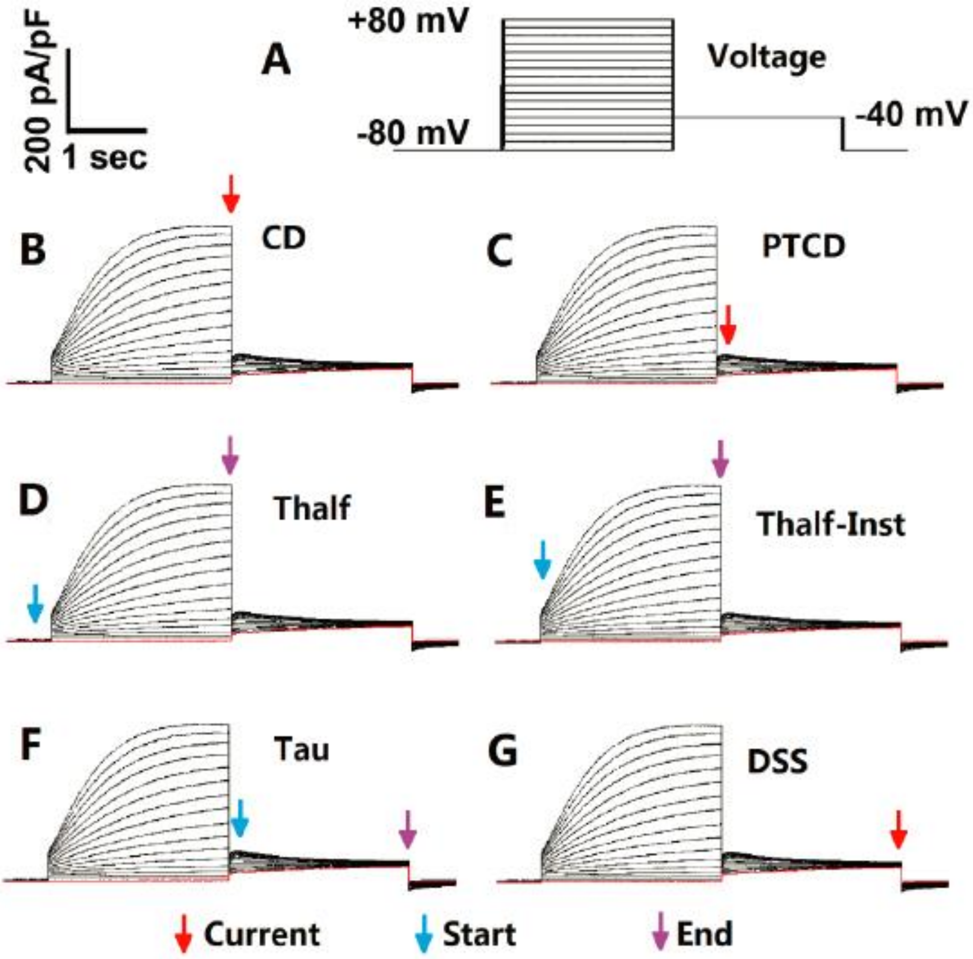

Figure S1. Patch clamp protocol and biomarkers calculation. **A**, voltage protocol; **B**, current density (CD); **C**, peak-tail current density (PTCD); **D**, activation thalf (Thalf); **E**, activation thalf-inst (Thalf-Inst); **F**, deactivation tau (Tau); **G**, deactivation steady state (DSS). Red arrows indicate the points where the current density signals were used to calculate the corresponding biomarkers, and the blue and purple arrows indicated the start and the end of the time periods during which the corresponding time constant biomarkers were calculated.

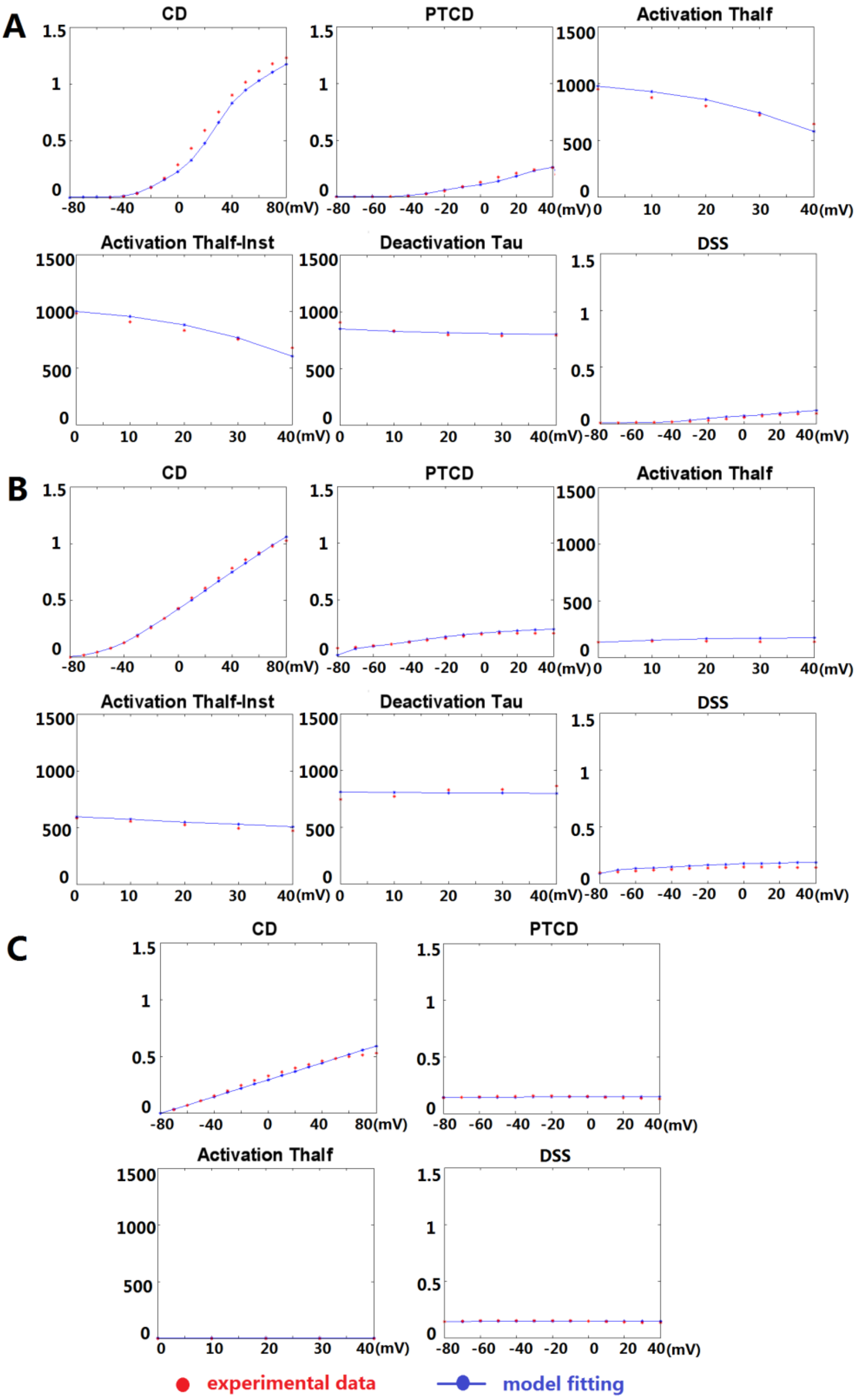

Figure S2. In silico fitting of the patch-clamp biomarkers from transiently transfected CHO-K1 cells: **A,** I_{Ks}-WT; **B,** I_{Ks}-HET; **C,** I_{Ks}-G229D.

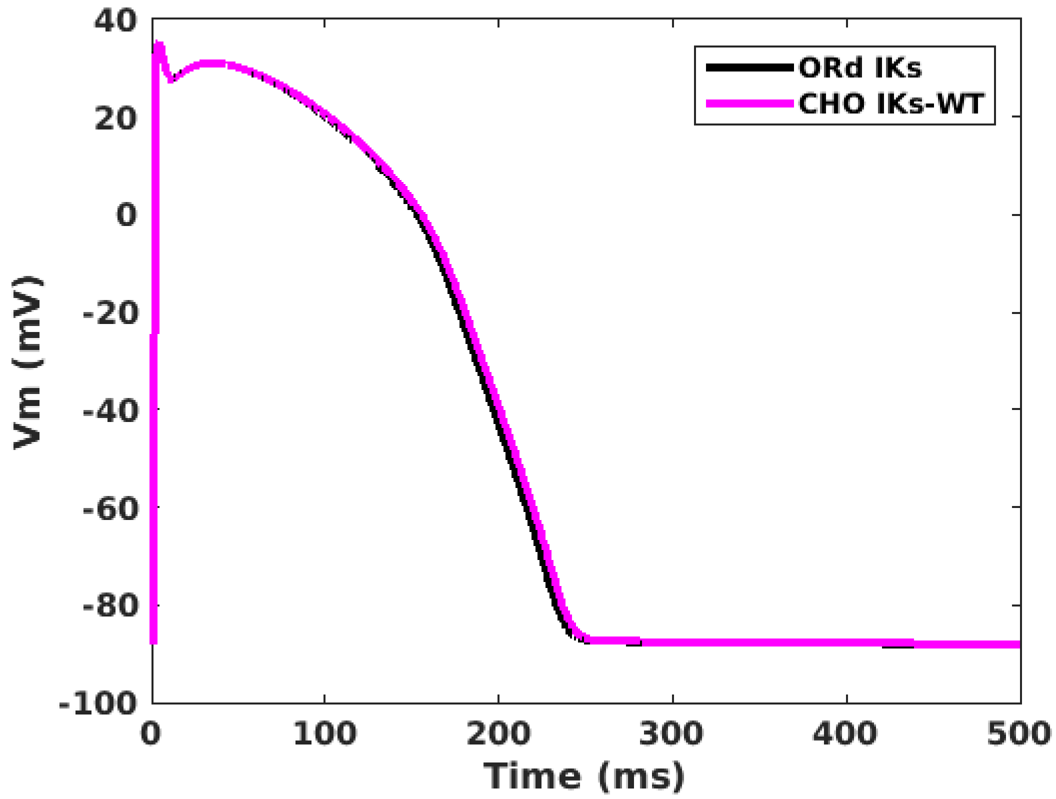

Figure S3. Comparison of the action potential traces using the ORd original I_{Ks} and the CHO-K1 (CHO) I_{Ks}-WT fitting.

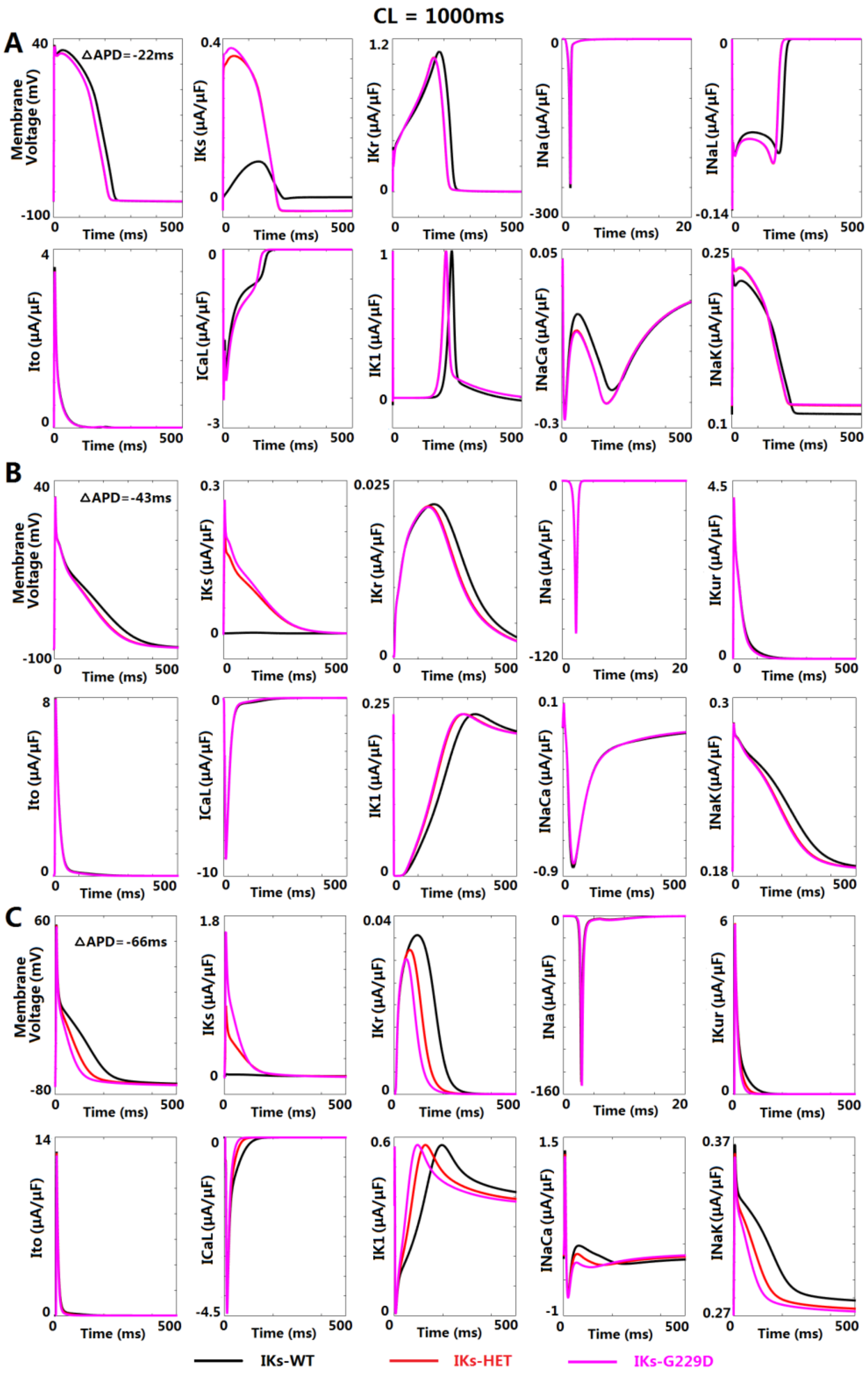

Figure S4. In-silico modelling of the effects of KCNQ1-G229D in the ORd human ventricular model (**A**), Grandi human atrial model (Grandi et al. 2011) (**B**) and Maleckar human atrial cell model (Maleckar et al. 2009) (**C**).

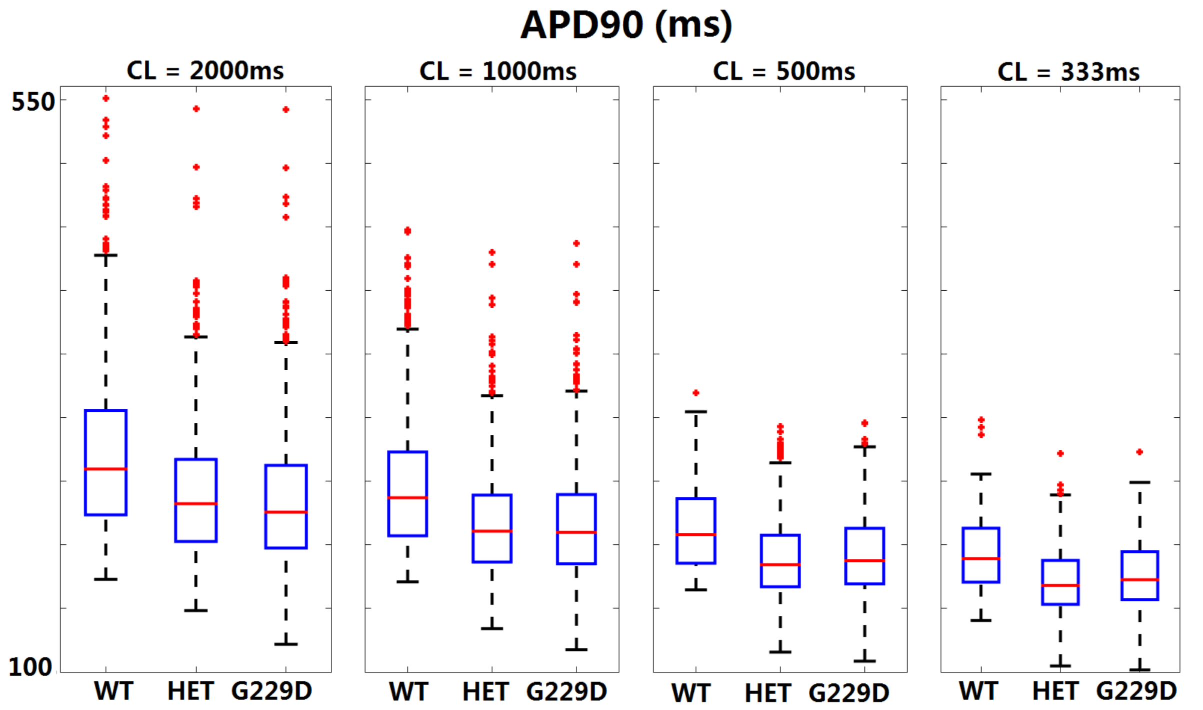

Figure S5. Distribution of APD_{90} in the presence or absence of the G229D mutation under four pacing rates in the ORd population of models. Red points indicate extreme values that lie more than 1.5 times the interquartile range away from the top (the 75th percentile) or bottom (the 25th percentile) of the box.

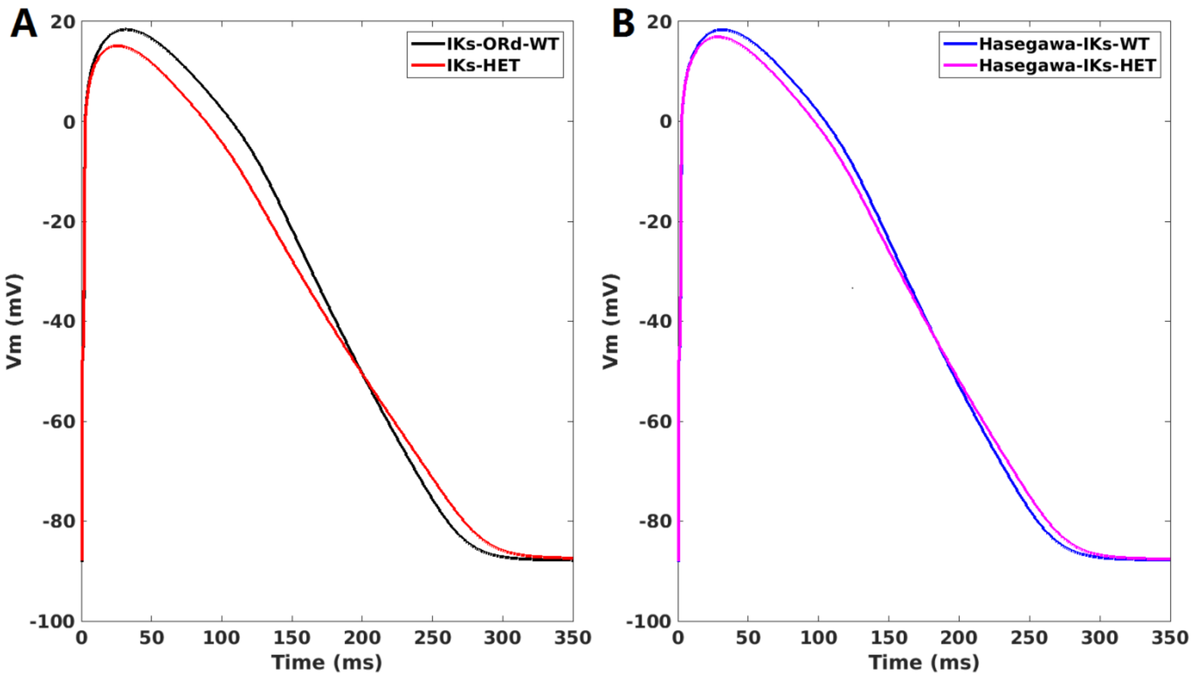

Figure S6. A representative model which displayed APD prolongation under I_{Ks}-HET in our simulations (**A**) also induced APD prolongation using the I_{Ks}-HET formulation reported by (Hasegawa et al. 2014) (CL=1000ms) (**B**).

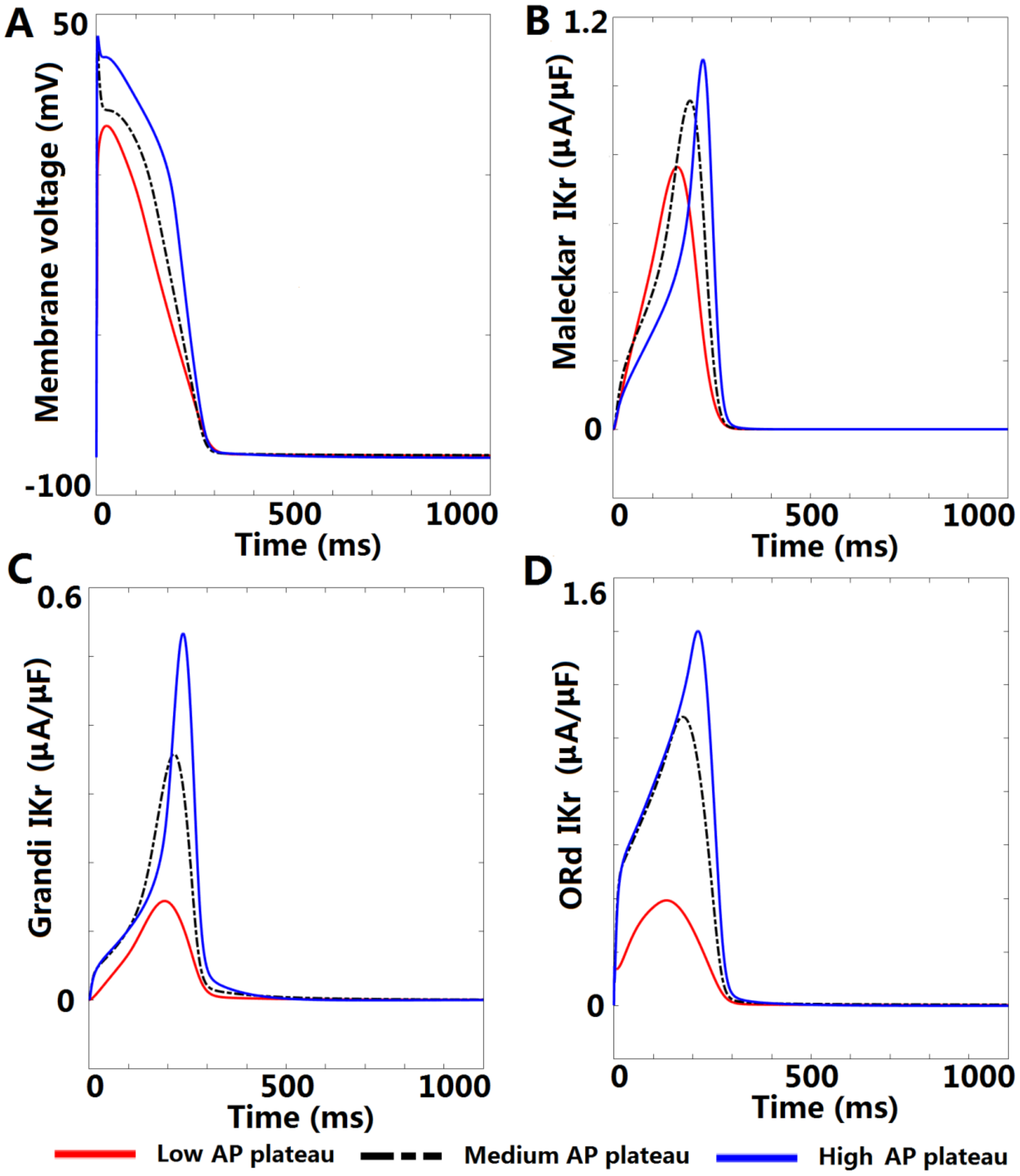

Figure S7. Action potential clamp simulation on different human I_{Kr} models. **A,** membrane voltage of three different plateaus used in the AP clamp simulation. I_{Kr} current under AP clamp traces in Maleckar model (**B**), Grandi model (**C**) and ORd model (**D**).

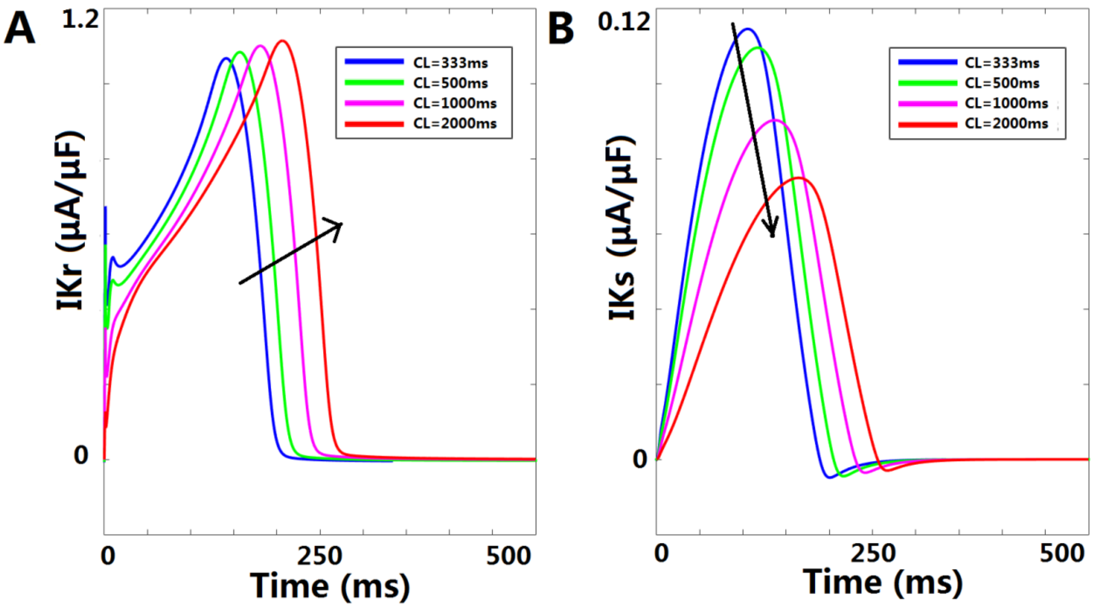

Figure S8. Rate dependency of I_{Kr} **(A)** and I_{Ks} **(B)** in the ORd model after pacing 1000 beats**.** Arrows indicate slower pacing.

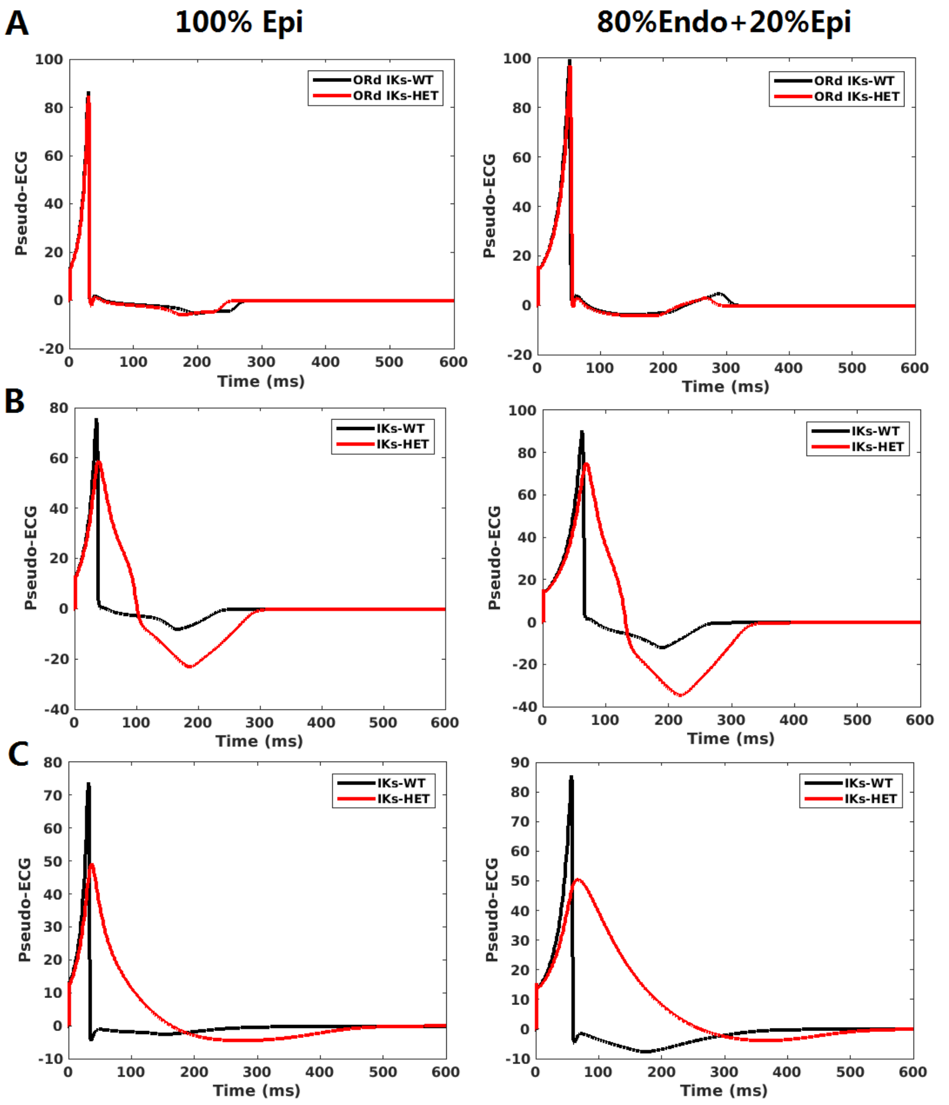

Figure S9. Comparison of the pseudo-ECGs from homogeneous epicardial fibers and transmural fibers (80% Endocardial + 20% Epicardial) in three representative examples. (**A**) ORd model; (**B**) The representative model that showed slower conduction in Figure 6C; (**C**) The representative model that showed depolarization abnormality in Figure 6D.

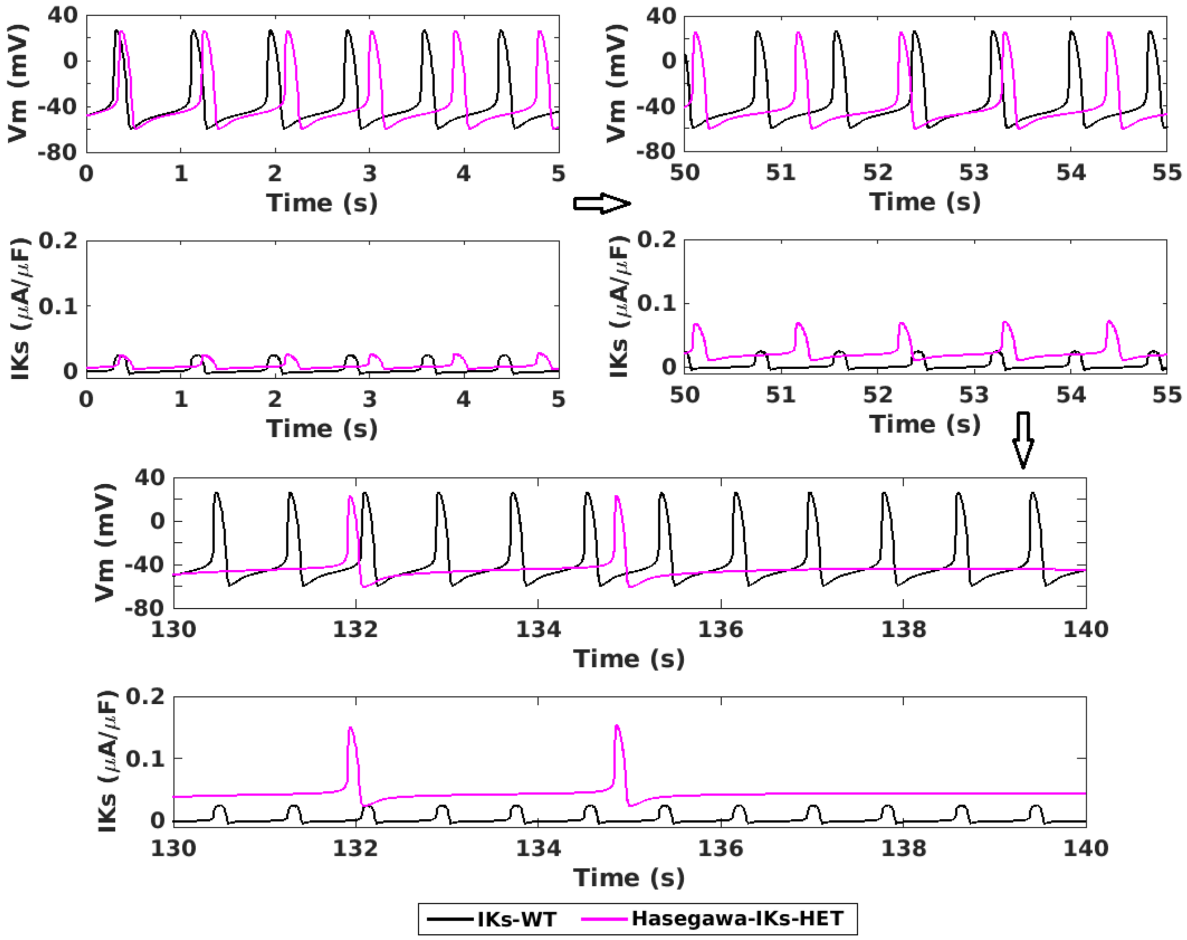

Figure S10. The I_{Ks}-HET formulation reported by (Hasegawa et al. 2014) also results in sinus node dysfunction.

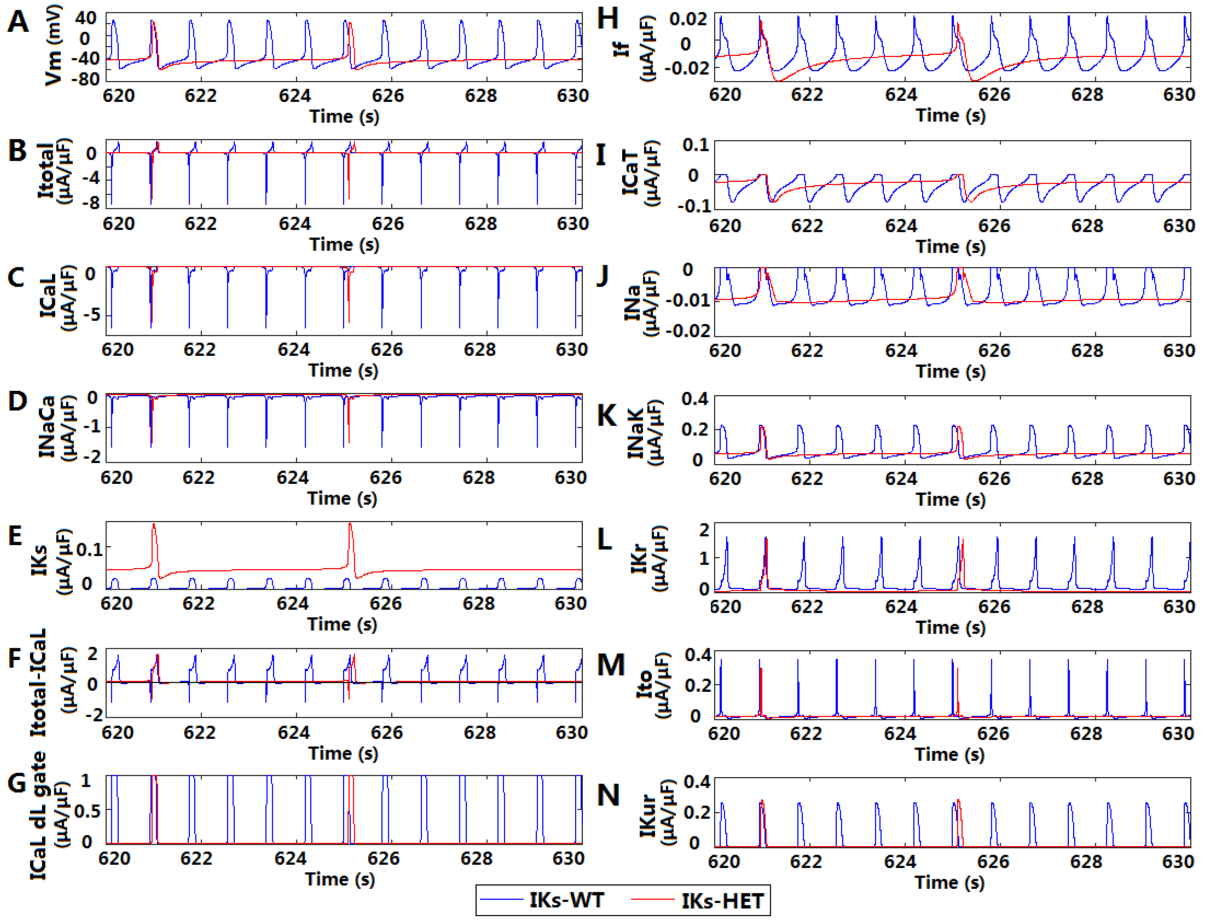

Figure S11. Contribution of sarcolemmal currents to the spontaneous pacemaking process. During the upstroke phase of membrane potential (**A**), I_{CaL} (**C**) and I_{NaCa} (**D**) contributed most to the negative peak of total current (I_{total}, **B**), and the net current excluding I_{CaL} (**F**) showed similar negative magnitude as I_{NaCa}. I_{CaL} activation gate dL (**G**) was rapidly activated during upstroke when I_{NaCa} reaches maximum activation (**D**). I_f (**H**), I_{CaT} (**I**), I_{Na} (**J**) provided inward currents for the slow diastolic depolarization process before upstroke, while I_{Ks}-HET (**E**), I_{NaK} (**K**) provided outward currents. I_{Kr} (**L**), I_{to} (**M**) and I_{Kur} (**N**) contributed very little to the diastolic depolarization process and activated during the upstroke phase to promote repolarization.

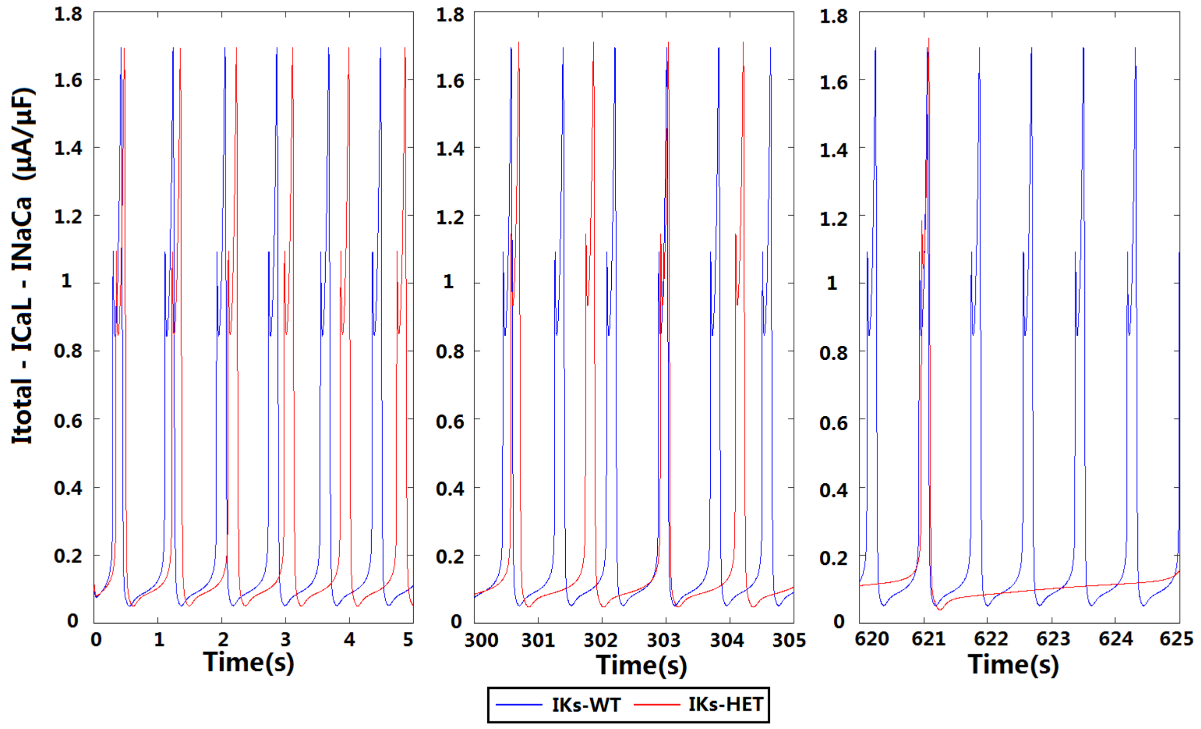

Figure S12. Sum of net sarcolemmal currents excluding I_{NaCa} and I_{CaL}. For both I_{Ks}-WT and I_{Ks}-HET, and from the initiation (Left, time=1->5s), to bradycardia (Middle, time=300->305s) and the final spontaneous beat before pacemaking failure (Right, time=620->625s), the total net current excluding I_{NaCa} and I_{CaL} was always outward (positive).

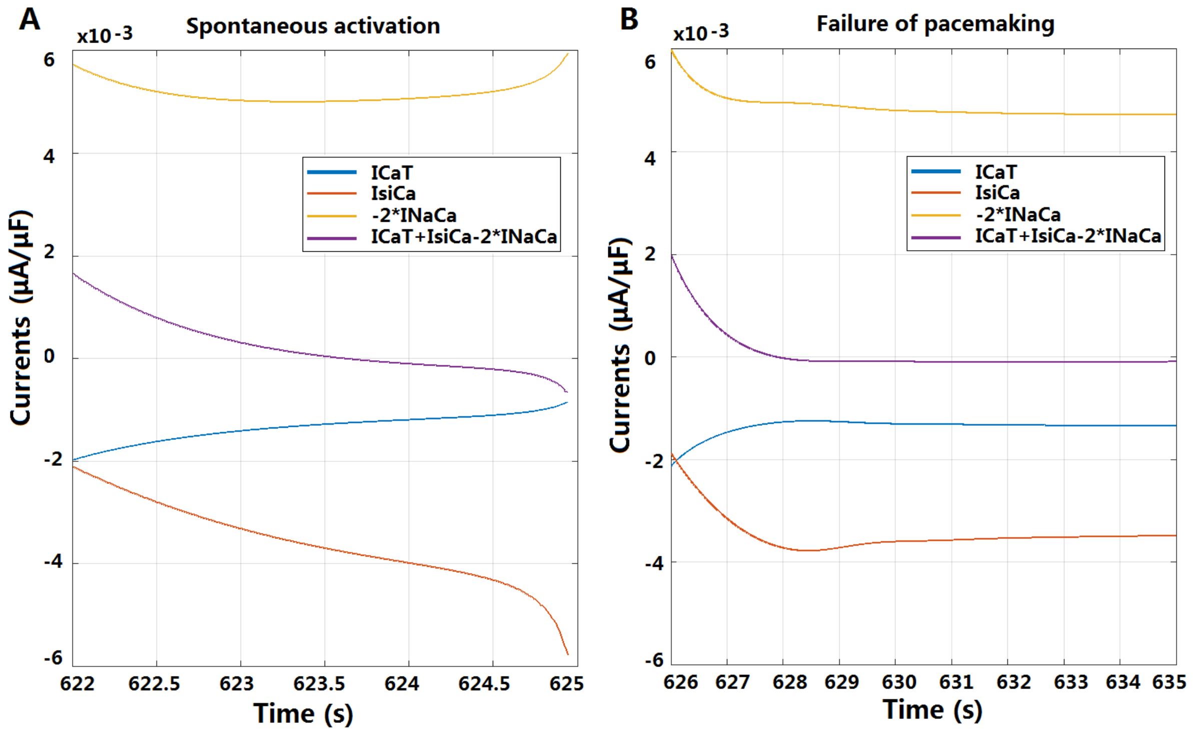

Figure S13. Contribution of I_{CaT}, I_{siCa} (calcium component of I_{CaL}) and I_{NaCa} to Ca_{sub}. Ca_{sub} accumulation rate was related to $-(\frac{I_{siCa}+I_{CaT}-2\times I_{NaCa}}{2\times F\times V_{sub}})$. Therefore, negative magnitudes of these currents corresponded to the accumulation of Ca_{sub}.

Supplementary References

Grandi, E., S. V. Pandit, N. Voigt, A. J. Workman, D. Dobrev, J. Jalife, and D. M. Bers. 2011. 'Human atrial action potential and Ca2+ model: sinus rhythm and chronic atrial fibrillation', *Circ Res*, 109: 1055-66.

Hasegawa, K., S. Ohno, T. Ashihara, H. Itoh, W. G. Ding, F. Toyoda, T. Makiyama, H. Aoki, Y. Nakamura, B. P. Delisle, H. Matsuura, and M. Horie. 2014. 'A novel KCNQ1 missense mutation identified in a patient with juvenile-onset atrial fibrillation causes constitutively open IKs channels', *Heart Rhythm*, 11: 67-75.

Maleckar, M. M., J. L. Greenstein, W. R. Giles, and N. A. Trayanova. 2009. 'K+ current changes account for the rate dependence of the action potential in the human atrial myocyte', *Am J Physiol Heart Circ Physiol*, 297: H1398-410.

O'Hara, T., L. Virag, A. Varro, and Y. Rudy. 2011. 'Simulation of the undiseased human cardiac ventricular action potential: model formulation and experimental validation', *PLoS Comput Biol*, 7: e1002061.
